# Gonadotropin-Releasing Hormone (GnRH) Receptor Structure and GnRH Binding

**DOI:** 10.3389/fendo.2017.00274

**Published:** 2017-10-24

**Authors:** Colleen A. Flanagan, Ashmeetha Manilall

**Affiliations:** ^1^Faculty of Health Sciences, School of Physiology, University of the Witwatersrand, Johannesburg, South Africa

**Keywords:** gonadotropin-releasing hormone receptor, G protein-coupled receptor, receptor structure, receptor activation, ligand binding

## Abstract

Gonadotropin-releasing hormone (GnRH) regulates reproduction. The human GnRH receptor lacks a cytoplasmic carboxy-terminal tail but has amino acid sequence motifs characteristic of rhodopsin-like, class A, G protein-coupled receptors (GPCRs). This review will consider how recent descriptions of X-ray crystallographic structures of GPCRs in inactive and active conformations may contribute to understanding GnRH receptor structure, mechanism of activation and ligand binding. The structures confirmed that ligands bind to variable extracellular surfaces, whereas the seven membrane-spanning α-helices convey the activation signal to the cytoplasmic receptor surface, which binds and activates heterotrimeric G proteins. Forty non-covalent interactions that bridge topologically equivalent residues in different transmembrane (TM) helices are conserved in class A GPCR structures, regardless of activation state. Conformation-independent interhelical contacts account for a conserved receptor protein structure and their importance in the GnRH receptor structure is supported by decreased expression of receptors with mutations of residues in the network. Many of the GnRH receptor mutations associated with congenital hypogonadotropic hypogonadism, including the Glu^2.53(90)^ Lys mutation, involve amino acids that constitute the conserved network. Half of the ~250 intramolecular interactions in GPCRs differ between inactive and active structures. Conformation-specific interhelical contacts depend on amino acids changing partners during activation. Conserved inactive conformation-specific contacts prevent receptor activation by stabilizing proximity of TM helices 3 and 6 and a closed G protein-binding site. Mutations of GnRH receptor residues involved in these interactions, such as Arg^3.50(139)^ of the DRY/S motif or Tyr^7.53(323)^ of the N/DPxxY motif, increase or decrease receptor expression and efficiency of receptor coupling to G protein signaling, consistent with the native residues stabilizing the inactive GnRH receptor structure. Active conformation-specific interhelical contacts stabilize an open G protein-binding site. Progress in defining the GnRH-binding site has recently slowed, with evidence that Tyr^6.58(290)^ contacts Tyr^5^ of GnRH, whereas other residues affect recognition of Trp^3^ and Gly^10^NH_2_. The surprisingly consistent observations that GnRH receptor mutations that disrupt GnRH binding have less effect on “conformationally constrained” GnRH peptides may now be explained by crystal structures of agonist-bound peptide receptors. Analysis of GPCR structures provides insight into GnRH receptor function.

## Introduction

Gonadotropin-releasing hormone (GnRH) regulates reproduction by binding and activating GnRH receptors on pituitary gonadotrope cells, which synthesize and secrete the gonadotropins, LH, and FSH. The gonadotropins act on the gonads to stimulate gametogenesis, gonadal cell proliferation, and production of the gonadal steroids. GnRH secretion is suppressed during childhood and increases at puberty, when increased production of gonadotropins and gonadal steroids initiate sexual development. Disruption of GnRH receptor function disrupts reproduction and mutations of the GnRH receptor gene disrupt or delay pubertal development, resulting in congenital hypogonadotropic hypogonadism (cHH) (1, 2). This central role in regulation of reproduction has made the GnRH receptor a target for treatment of infertility and of sex steroid-dependent hyperplasias, including uterine fibroids, endometriosis and prostatic cancer, where gonadal steroid production is decreased by administration of GnRH antagonists or high doses of GnRH agonists, which downregulate receptor expression (3–5). Agonist binding to the GnRH receptor activates the G_q/11_ family of heterotrimeric G proteins. Activated GTP-bound Gα_q/11_ subunits activate phospholipase Cβ, which catalyzes production of the second messengers diacylglycerol and inositol trisphosphate, which initiate the cellular signaling pathways that culminate in gonadotropin synthesis and secretion (3, 6, 7). Although the GnRH receptor is also reported to transiently activate G_s_ proteins in the LβT2 gonadotrope cell line (3, 8, 9) and inhibit cell growth *via* the inhibitory G_i_ proteins, no direct GnRH receptor activation of Gα_i_ or Gα_s_ could be shown in a range of cell lines (10–12) and it has been proposed that GnRH-stimulated activation of G_i_ or G_s_ proteins may be downstream of activation of the G_q/11_ proteins (6, 12). The mammalian (type 1) GnRH receptor does not activate β-arrestin-dependent signaling (3, 6, 7, 13), suggesting that all effects of the GnRH receptor may be mediated by activation of G_q/11_ proteins.

The GnRH receptor belongs to the G protein-coupled receptor (GPCR) family, which constitutes the largest family of membrane proteins in the human genome (14, 15). The GPCRs regulate physiological systems ranging from vision and olfaction through neurotransmission and immunology in addition to endocrine systems. Physiological ligands that activate GPCRs range from cations (Ca^2+^), small molecule neurotransmitters and immune modulators to peptide and protein hormones, cytokines and even light, which changes the 11-*cis*-retinal prosthetic group of rhodopsin from a covalently bound inverse agonist (an antagonist that actively stabilizes inactive receptor conformations) to an agonist. In spite of their diverse physiological functions and ligands, all GPCRs share a common molecular function, which consists of transducing an extracellular signal across a biological membrane *via* a change in receptor protein conformation (16–18). This conserved function is supported by a conserved protein structure that consists of an extracellular amino-terminus, a bundle of seven membrane-spanning α-helical segments connected by three intracellular and three extracellular loops and a cytoplasmic carboxy-terminus (16, 19, 20). No crystal structure of the GnRH receptor has yet been reported, but much can be learned about its structure and how it conveys the extracellular GnRH-binding signal to intracellular signaling pathways by studying the structures of related GPCRs that have been crystallized and combining this with biochemical studies. This review will focus on understanding of the structure of the GnRH receptor and ligand binding that has arisen since the last major review (13) with emphasis on the application of recently described GPCR structures and how these may inform mechanisms of GnRH receptor structure, activation and ligand binding.

## Primary Structures of GnRH Receptors

Based on conserved amino acid sequence features (Table 1), the GnRH receptor is a class A GPCR. Class A is the largest and best-studied class of GPCR proteins and includes rhodopsin, adrenergic and other monoamine neurotransmitter receptors and many peptide and protein-binding receptors. The membrane-spanning segments of GPCRs are most conserved, whereas the loops and termini are more variable (19). To facilitate comparison of amino acid residues of the GnRH receptor with equivalent residues of other class A GPCRs, the Ballesteros and Weinstein numbering system (21) will be used. Residues are numbered relative to the most conserved residue in each transmembrane (TM) segment, which is designated .50, preceded by the TM segment number and followed, where relevant, by the amino acid sequence number in the receptor in parenthesis. For example Asp^319^ of the human GnRH receptor is designated Asp^7.49(319)^, because it immediately precedes the most conserved residue in TM7, Pro^7.50(320)^. The equivalent residue of the mouse receptor is Asp^7.49(318)^.

**Table 1:** Highly conserved amino acid residues and motifs in class A GPCRs and equivalent residues in type 1 and type 2 GnRH receptors.

Conserved GPCR residue or motif	Function in GPCRs	Reference	Residue in human type 1 GnRH receptor	Residue in type 2 GnRH receptors	Function in GnRH receptors	Reference
Asn^1.50^	Part of the conformation-independent conserved interhelical network Part of the water-mediated polar networks	(19, 20) (22)	Asn^1.50(53)^	Asn^1.50^	Structural^a^	(23)

Asp^2.50^	Part of the conformation-independent conserved interhelical network Part of the water-mediated polar networks Binding of Na^+^	(19, 20) (22) (24)	Asn^2.50(87)^	Asp^2.50^	Structural	(23, 25)

Asp^3.49^–Arg^3.50^–Tyr^3.51^ (DRY)	Part of the ionic lock Interacts with G proteins	(26–28)	Asp^3.49(138)^–Arg^3.50(139)^–Ser^3.51(140)^ (DRS)	Asp^3.49^–Arg^3.50^–Xaa^3.51^ (DRx)	Structural and activation of cellular signaling	(29, 30)

Trp^4.50^	Part of the conserved conformation-independent interhelical network	(19, 20)	Trp^4.50(164)^	Trp^4.50^		

Pro^5.50^	Part of the transmission switch	(22, 26, 31, 32)	Pro^5.50(223)^	Pro^5.50^		

Cys^6.47^–Trp^6.48^–x–Pro^6.50^–Tyr^6.51^ (CWxPY)	Part of the conformation-independent conserved interhelical network	(19, 20)	Cys^6.47(279)^–Trp^6.48(280)^–Thr^6.49(281)^–Pro^6.50(282)^–Tyr^6.51(283)^	Cys^6.47^–Trp^6.48^–Thr^6.49^–Pro^6.50^–Tyr^6.51^	Structural and ligand-binding affinity.	(34–38)
	Part of the conserved intramolecular water-mediated polar networks	(22)				
	Forms an exaggerated kink that opens the G protein-binding pocket when TM6 rotates	(33)				

Asn^7.49^–Pro^7.50^–x–x–Tyr^7.53^ (NPxxY)	Part of the conformation-independent conserved interhelical network	(19, 20)	Asp^7.49(319)^–Pro^7.50(320)^–Leu^7.51(321)^–Ile^7.52(322)^–Tyr^7.53(323)^ (DPxxY)	Asp^7.49^–Pro^7.50^–x–x–Tyr^7.53^ (DPxxY)	Structural, possible Na^+^ counter-ion, activation of cellular signaling	(23, 25, 36, 39)
	Part of the conserved intramolecular water-mediated polar networks	(22)				
	Forms conformation-specific interhelical interactions	(17)				

*A brief summary of the key functions of highly conserved residues revealed by structures of class A GPCRs is provided with a listing of the functions of equivalent GnRH receptor residues based on functional (site-directed mutagenesis) studies*.

*^a^Structural effects relate to effects on cell surface expression of mutant receptors. Prior to development of technology to “rescue” expression, mutants that were not expressed could not be studied further*.

This review will focus on the mammalian type 1 GnRH receptor, which is characterized by absence of a cytoplasmic carboxy-terminal tail (13, 40) that accounts for the lack of arrestin-dependent desensitization, internalization, and signaling. Many systems of nomenclature have been used for GnRH receptor subtypes, largely because of the unclear relationship between the tailless mammalian receptors and the other GnRH receptors, all of which have carboxy-terminal tails (13, 41, 42). The discovery that some lower vertebrates have tailless GnRH receptors that are structurally and functionally similar to mammalian receptors (43) has now provided some consensus (40, 44–46). All of the tailless GnRH receptors are designated type 1 and all of the tailed GnRH receptors, type 2.

Human GnRH receptors have all of the highly conserved Ballesteros and Weinstein reference residues, except for the acidic Asp^2.50^ in TM2, which is substituted with uncharged Asn (Table 1; Figure S1 in Supplementary Material). Mutation of Asn^2.50(87)^ to the normal Asp disrupted GnRH receptor expression (23, 25), confirming the functional importance of the substitution. The type 1 GnRH receptors also have variations of the highly conserved amino acid sequence motifs. In TM7 the NPxxY motif (Asn^7.49^-Pro^7.50^-x-x-Tyr^7.53^ where x represents any amino acid) is changed to **D**PxxY (**Asp**^7.49^-Pro^7.50^-Leu^7.51^-Ile^7.52^-Tyr^7.53^). Mutation of Asp^7.49^ to Asn reversed the disruption of GnRH receptor expression caused by mutation of Asn^2.50^ to Asp, suggesting these resides might be close to each other in the three-dimensional structures of class A GPCRs (25). The CWxPY motif in TM6 is preserved as Cys^6.47^-Trp^6.48^-Thr^6.49^-Pro^6.50^-Tyr^6.51^, whereas the DRY motif at the cytosolic end of TM3 is DRS (Asp^3.49^-Arg^3.50^-Ser^3.51^) (Table 1; Figure S1 in Supplementary Material).

Type 1 GnRH receptors have a Glu^2.53(90)^ residue in TM2, which has risen to prominence because a cHH-associated Glu^2.53(90)^Lys mutation disrupts membrane expression of the receptor, but treatment with a pharmacoperone [small-molecule membrane-permeable GnRH receptor antagonists that act as templates for folding of nascent receptor proteins (34, 35, 47)] rescues expression of the mutant receptor, both *in vitro* and in knock-in transgenic mice (34, 47, 48). In other class A GPCRs the equivalent residue is mostly large and hydrophobic (Leu, Val, or Phe) (49) and is Ile^2.53^, Val^2.53^, or Met^2.53^ in type 2 GnRH receptors (13, 43), suggesting that the carboxyl side chain of Glu^2.53(90)^ may not be required.

The functional importance of the highly conserved Tyr^5.58^ residue was revealed by crystal structures of active rhodopsin (33, 50). Type 1 GnRH receptors have Asn^5.58^, but all tailed GnRH receptors have the conserved Tyr^5.58^ (13, 40, 43). In most class A GPCRs a conserved large aliphatic amino acid, Ile^3.40^, forms part of a group of conserved residues referred to as the “core triad” (22) or “transmission switch” (26, 31, 32), which changes configuration during receptor activation. GnRH receptors have a small Ala^3.40(129)^ residue, which is also present in type 2 GnRH receptors.

## The Three-Dimensional Structure of the GnRH Receptor

Ligands interact with the variable extracellular half of GPCR molecules. The membrane-spanning domain conveys the ligand-binding signal to the cytosolic surface of the receptor, which interacts with the G protein (20). In order to specifically transduce an agonist signal across a cellular membrane, a GPCR must exist in a silent state that does not activate G proteins. Once agonist binds, the receptor must undergo transition to a state that binds and activates G proteins located on the opposite side of the membrane. Thus, agonist ligands, such as GnRH, can be thought of as allosteric activators of GPCRs, enabling them to catalyze G protein activation (16, 18, 51).

Current theories of receptor activation posit that GPCRs exist in an equilibrium of inactive “R” and activated “R*” conformations, with the equilibrium balanced toward the R conformations in the absence of ligand. The R conformations cannot activate G proteins, are stabilized by binding of inverse agonist (antagonist) ligands and have low affinity for agonist ligands. In contrast, R* conformations bind and activate G proteins, have high affinity for agonist ligands and are stabilized by binding of agonists and/or G proteins (16, 52–54). Thus, agonist binding induces or stabilizes one or more active GPCR conformation(s), which activate G protein signaling. Similarly, G protein binding increases the binding affinity of the receptor for agonist (16, 54, 55). Until recently, the structural correlates of the R and R* conformations were unknown, but a flurry of technical innovations has recently allowed X-ray crystallographic determination of the structures of rhodopsin and then other GPCRs. Initial structures were bound to inverse agonists and thus represent inactive R conformations. These were followed by crystal structures of agonist-bound GPCRs that were partially active, whereas, in most cases, cocrystallization of agonist-bound receptors with a G protein or a G protein mimetic (antibody or truncated G protein) was required to achieve fully active GPCR structures (16, 27, 31, 32, 56). The GPCR crystal structures reveal the differences between the R and R* conformations, although they do not provide dynamic information about the activation process. We will attempt to use information from the structures of class A GPCRs that have been crystallized to understand GnRH receptor structure.

### Conformation-Independent Intramolecular Interactions

G protein-coupled receptor crystal structures show that the three-dimensional structures of GPCRs are more conserved than the amino acid sequences (15, 19, 20, 26, 57). The convergence of diverse amino acid sequences to a common structure allowed considerable plasticity in the evolutionary development of the diverse GPCR family (17, 19, 57). The crystal structures have defined the relative positions of the known highly conserved amino acids and of the conserved amino acid sequence motifs and shown that the highly conserved Pro^5.50^, Pro^6.50^, and Pro^7.50^ residues in TM5, TM6, and TM7 cause bends in the α-helices that are not classical proline kinks (32, 58, 59).

#### The TM6 Proline Kink

The exaggerated bend angle around Pro^6.50^, in the CWxPY motif in TM6, is stabilized by a water molecule that makes hydrogen bonds to the Cys^6.47^ and Tyr^6.51^ residues of the CWxPY motif and to a residue in TM7 in GPCR structures (33, 58, 60–62). The importance of this structure in the GnRH receptor is supported by no less than three cHH-associated mutations of the CWxPY motif. The Pro^6.50(282)^Arg mutation of the GnRH receptor completely disrupts receptor function, which cannot be recovered by pharmacoperone treatment (36), showing that the proline kink is essential for GnRH receptor expression. The Cys^6.47(279)^Tyr mutant GnRH receptor and a laboratory-produced Cys^6.47(279)^Ala mutant showed no measurable GnRH binding and severely decreased cellular responses to GnRH stimulation that were recovered after pharmacoperone treatment of cells transfected with the mutant receptor (1, 35, 63, 64). This suggests that the Cys^6.47(279)^ mutations disrupt receptor folding during biosynthesis, but the rescue shows that its effect is less destructive than mutating the Pro^6.50(282)^. A Tyr^6.51(283)^His GnRH receptor mutation causes cHH and results in no measurable function *in vitro* (65).

#### Conformation-Independent Interhelical Contacts Form a Conserved Scaffold

The overall GPCR fold (the relative positions of the seven TM segments) is stabilized by ~200–260 non-covalent intramolecular contacts (hydrogen bonds, van der Waals interactions, etc.) and by a network of hydrogen-bonded water molecules in the interior of the TM domain. Depending on methodology, 24–40 interhelical contacts between topologically equivalent loci (positions) are present in all active and inactive class A GPCR structures (17, 19, 20). These conserved conformation-independent interhelical contacts determine the overall GPCR structure, forming a conserved “scaffold,” on which conformational changes can occur. Some of the conserved interhelical contacts are required for protein folding and insertion into the membrane during biosynthesis. The conserved interhelical contacts involve many of the residues that are highly conserved in class A GPCRs (Table S1 in Supplementary Material) but also involve residues in topologically equivalent loci, where the amino acids are not conserved (19, 20, 57). Most of the conserved conformation-independent interhelical interactions are located toward the central and cytoplasmic side of the TM domain (20) (Figure S2 in Supplementary Material), consistent with the emerging recognition that these are the areas in which GPCR structure is most conserved, whereas the extracellular side of the TM barrel is less conserved, because of the need to accommodate diverse ligands (18).

Gonadotropin-releasing hormone receptor residues topologically equivalent to the residues in conserved conformation-independent interhelical contacts are listed in Table S1 in Supplementary Material, with the predicted interhelical contacts and effects of previously reported mutations of the residues on GnRH receptor expression and function. Many mutations result in undetectable receptor function, consistent with disruption of cell surface GnRH receptor expression or severe misfolding of the receptor protein. The disruption of functional receptor expression confirms the importance of the residues for GnRH receptor structure and indicates that the conserved interhelical contacts constitute part of the GnRH receptor structure, similar to their roles in other GPCRs.

The complete disruption of GnRH receptor expression when Asn^2.50(87)^ in TM2 was substituted with Ala or Asp (Table S1 in Supplementary Material) supports a role for the Asn^2.50(87)^ residue in stabilizing GnRH receptor structure. However, GPCR crystal structures show that Asp^2.50^ makes conserved contacts with residues in TM1 and TM7, but it is only connected to Asn^7.49^ *via* the water-mediated hydrogen bond network (19, 58). Based on the conserved structural scaffold, Asn^2.50(87)^ of the GnRH receptor contacts Asn^1.50(53)^ and Pro^7.46(316)^, whereas Asp^7.49(319)^ does not form any conserved conformation-independent contacts (Table S1 in Supplementary Material).

#### cHH-Associated GnRH Receptor Mutations Affect Conserved Conformation-Independent Interhelical Contacts

Many cHH-associated GnRH receptor mutations involve residues that constitute conserved interhelical contacts in the crystallized GPCR structures. These include the Glu^2.53(90)^Lys, Glu^2.53(90)^Asp, Ala^4.57(171)^Thr, Cys^6.47(279)^Tyr, Tyr^6.51(283)^His, Tyr^6.52(284)^Cys, Pro^7.50(320)^Arg, and Tyr^7.53(323)^Cys mutations. Most cHH-associated mutant receptors are poorly expressed *in vitro* (Table S1 in Supplementary Material), consistent with the mutations disrupting the structural scaffold of the receptor. Disruption of interhelical contacts would destabilize receptor protein folding, resulting in fewer correctly folded receptor molecules being transported to the cell membrane or decreased residence time of less stable receptor proteins once they get to the cell membrane. Pharmacoperones act as templates for folding of nascent receptor proteins, stabilizing biosynthesis, and increasing protein expression (34, 35, 47). In most cases, pharmacoperone treatment of cells transfected with cHH-associated mutant GnRH receptors, enhanced mutant receptor expression (Table S1 in Supplementary Material) and the “rescued” receptors showed wild type-like function. This supports a role for the mutated residues in stable folding and cell surface expression of the GnRH receptor (34, 35, 47).

The late Michael Conn’s laboratory and others proposed that the Glu^2.53(90)^ side chain of the GnRH receptor forms a interhelical salt-bridge with Lys^3.32(121)^ in TM3 and that the cHH-associated Glu^2.53(90)^Lys mutation disrupted receptor biogenesis and folding by breaking this salt bridge (66–69). Although Glu^2.53(90)^ is conserved in type 1 GnRH receptors, type 2 GnRH receptors and other class A GPCRs have large hydrophobic residues at position 2.53 (13, 43, 49, 57). The lack of conservation suggests that the acidic side chain of Glu^2.53(90)^ may not be necessary for type 1 GnRH receptor structure and that the effect of the Glu^2.53(90)^Lys mutation may result from disruptive effects of introducing Lys, rather than lack of Glu. This is supported by a mutation of Glu^2.53(90)^ to uncharged Gln, which had no effect on receptor function (70) and a conservative Glu^2.53(90)^Asp, which is associated with cHH (Table S1 in Supplementary Material). A Glu^2.53(90)^Ala mutation resulted in undetectable GnRH receptor function (68), but we have not found any report of the effect of the Glu^2.53(90)^Asp mutation, which would formally test the salt bridge hypothesis.

**Figure 1:**
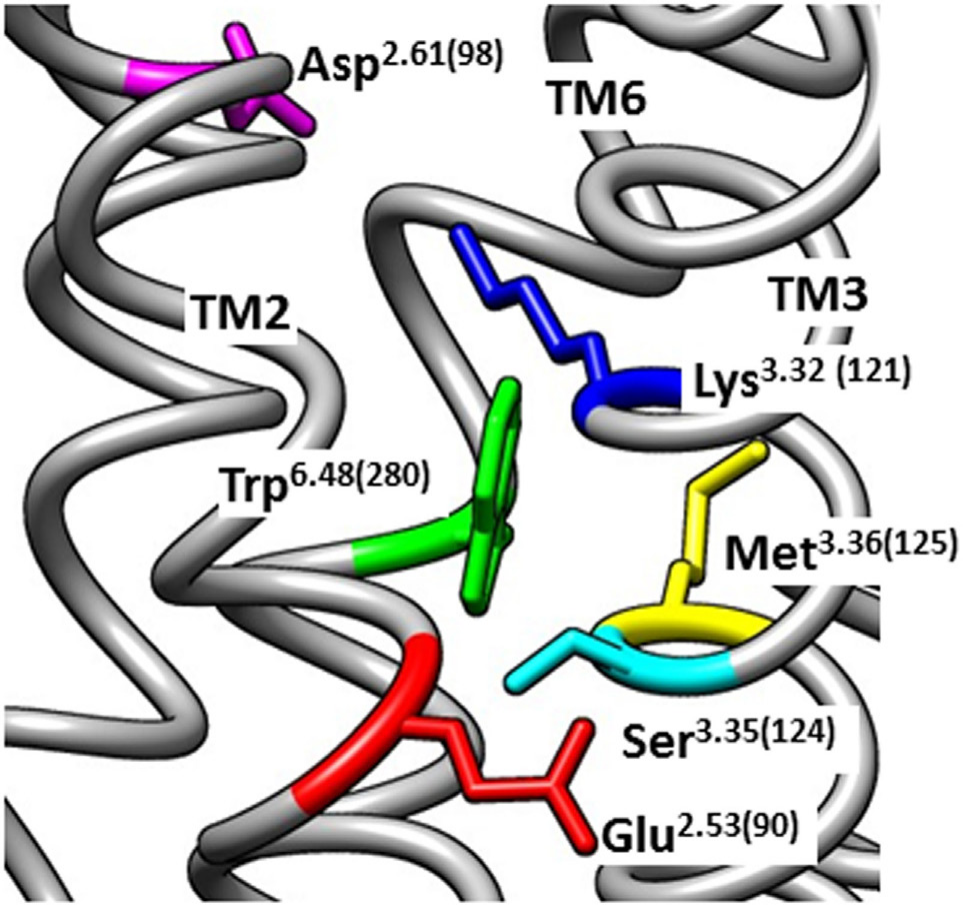
Homology model of the inactive human gonadotropin-releasing hormone (GnRH) receptor. The model was downloaded from the GPCRdb website (www.gpcrdb.org/structure/homology_models) (71) and viewed using the UCSF Chimera software package (72) to show the spatial positioning of Glu^2.53(90)^ (red) relative to the neighboring residues Ser^3.35(124)^ (light blue), Lys^3.32(121)^ (dark blue), Asp^2.61(98)^ (magenta), Met^3.36(125)^ (yellow), and Trp^6.48(280)^ (green). Chimera is developed by the Resource for Biocomputing, Visualization, and Informatics at the University of California, San Francisco (supported by NIGMS P41-GM103311).

The conserved interhelical contacts in GPCR structures predict that Glu^2.53(90)^ interacts with Ser^3.35(124)^ (Table S1 in Supplementary Material). Interaction of Glu^2.53(90)^ with Ser^3.35(124)^ is supported by an automated (unbiased) structural homology model of the GnRH receptor (71), which shows Glu^2.53(90)^ close to Ser^3.35(124)^, whereas Lys^3.32(121)^ points away, toward Asp^2.61(98)^ (Figure 1). A Ser^3.35(124)^Asp mutation resulted in undetectable binding, suggesting that the mutation caused receptor instability by introducing close apposition of two carboxyl side chains (37). Thus, it is likely that the cHH-associated Glu^2.53(90)^Lys mutation disrupts GnRH receptor expression by disrupting the conserved interhelical contact with the 3.35 locus, rather than disrupting a salt-bridge with Lys^3.32(121)^.

Another cHH-associated GnRH receptor mutant, Tyr^6.51(283)^His, also showed undetectable function *in vitro* (65). Since Tyr^6.51^ forms a conserved interhelical contact with the residue in the 7.39 locus, the Tyr^6.51(283)^His mutation may disrupt receptor structure by disrupting an interhelical contact of Tyr^6.51(283)^ with Phe^7.39(309)^ in TM7 of the GnRH receptor (Table S1 in Supplementary Material).

#### Role of Trp^6.48(280)^ of the CWxPY Motif in the Conserved Structural Scaffold of the GnRH Receptor

The Trp^6.48(280)^ residue in the CWxPY motif was proposed to directly contact the Trp^3^ residue of the GnRH peptide (69, 73). Systematic mutagenesis of Trp^6.48(280)^ to Ala, His, Ser, Gln, and Met disrupted GnRH receptor expression, as did mutation of Trp^6.48(279)^ of the rat GnRH receptor to Arg or Ser (74). However, once expression of the mutant receptors was recovered, using a pharmacoperone, the mutant receptors displayed unchanged ligand-binding affinity and signaling (37, 38, 73). This shows that Trp^6.48(280)^ does not directly contact GnRH, but is important for GnRH receptor structure. Trp^6.48(280)^ likely forms conserved interhelical contacts with Met^3.36(125)^ and Ala^7.42(312)^ of the GnRH receptor (Table S1 in Supplementary Material), which would be disrupted by the mutations.

### The Inactive Receptor Structure

**Figure 2:**
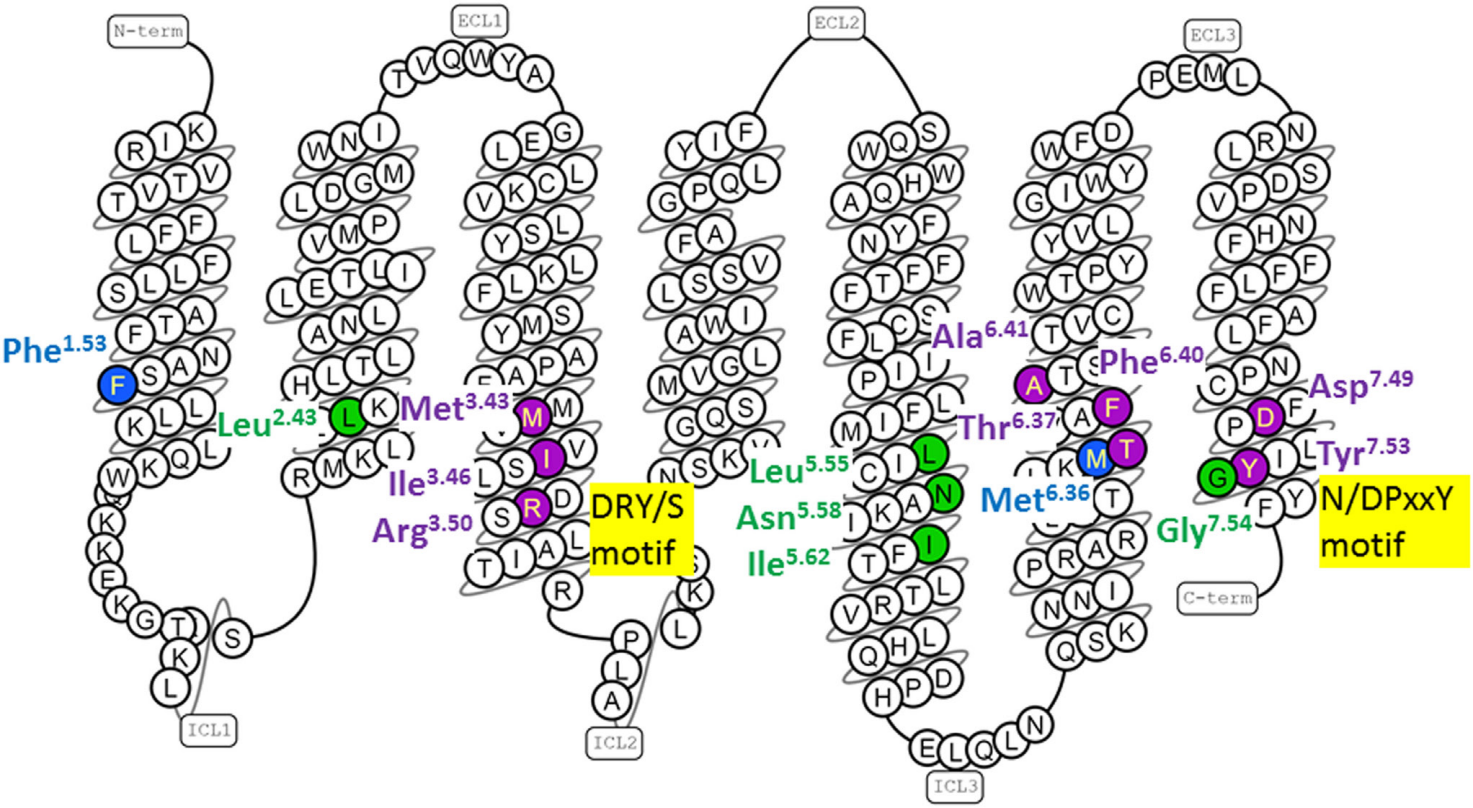
Human gonadotropin-releasing hormone (GnRH) receptor residues topologically equivalent to G protein-coupled receptor (GPCR) residues that form conserved conformation-specific interhelical contacts. The snake diagram of the GnRH receptor amino acid sequence was downloaded from GPCRdb (www.gpcrdb.org/structure/homology_models) (71). GnRH receptor residues topologically equivalent to residues that form conserved conformation-specific contacts (17, 19) only in inactive GPCR structures (blue); only in active GPCR structures (green) and with different partners in inactive and active GPCR structures (purple) are shown.

About half of the intramolecular interactions differ between the inactive and active GPCR structures. The GnRH receptor residues equivalent to the residues that form conserved conformation-specific interhelical contacts are listed in Table 2. These conformation-specific interhelical contacts depend on reassignment of the interacting amino acid pairs during the activation process (17, 19) (Figure 2). This section will describe the key features of the inactive GPCR structures and discuss the evidence for similar structural features in the GnRH receptor. The main features of inactive GPCR structures include a “closed” G protein-binding pocket, a water-mediated hydrogen bond network and a hydrophobic barrier separating the water network in the ligand-binding pocket from the G protein-binding pocket (17, 18, 22, 32, 33, 56, 75).

**Table 2:** GnRH receptor residues potentially involved in conserved conformation-specific interhelical contacts.

GnRH receptor residue	Inactive conformation-specific interhelical contacts	Active conformation-specific interhelical contacts	GnRH receptor mutations	Effects of mutations	Reference
Phe^1.53(56)^	Phe^1.53(56)^–Tyr^7.53(323)^				

Leu^2.43(80)^		Leu^2.43(80)^–Gly^7.54(324)^	Leu^2.43(80)^Ala	Decreased expression and decreased agonist potency	(76)

Met^3.43(132)^	Met^3.43(132)^–Phe^6.40(272)^ Met^3.43(132)^–Ala^6.41(273)^	Met^3.43(132)^–Asp^7.49(319)^Met^3.43(132)^–Tyr^7.53(323)^	Met^3.43(132)^Ala	Undetectable,^a^ expression, and signaling rescued by pharmacoperone^b^	(77)

Ile^3.46(135)^	Ile^3.46(135)^–Thr^6.37(269)^	Ile^3.46(135)^–Tyr^7.53(323)^	Ile^3.46(135)^Ala Ile^3.46(135)^Leu Ile^3.46(135)^Val	Undetectable Increased coupling efficiency Undetectable	(29)

Arg^3.50(139)^	Arg^3.50(139)^–Thr^6.37(269)^	Arg^3.50(139)^–Phe^6.40(272)^	Arg^3.50(139)^His Arg^3.50(139)^Lys Arg^3.50(139)^Gln Arg^3.50(139)^Ala Arg^3.50(139)^Cys	Undetectable, cHH, rescued by pharmacoperone Undetectable Increased expression uncoupled Decreased expression and coupling cHH, decreased expression, rescued by pharmacoperone, decreased coupling	(47, 78, 79) (29) (30)	

Leu^5.55(228)^		Leu^5.55(228)^–Ala^6.41(273)^			

Asn^5.58(231)^		Asn^5.58(231)^–Phe^6.40(272)^			

Ile^5.62(235)^		Ile^5.62(235)^–Thr^6.37(269)^			

Met^6.36(268)^	Met^6.36(268)^–Tyr^7.53(323)^				

Thr^6.37(269)^	Ile^3.46(135)^–Thr^6.37(269)^ Arg^3.50(139)^–Thr^6.37(269)^	Ile^5.62(235)^–Thr^6.37(269)^	Thr^6.37(269)^Met	cHH, undetectable	(80)

Phe^6.40(272)^	Met^3.43(132)^–Phe^6.40(272)^ Phe^6.40(272)^–Asp^7.49(319)^	Arg^3.50(139)^–Phe^6.40(272)^Asn^5.58(231)^–Phe^6.40(272)^	Phe^6.40(272)^Ala Phe^6.40(272)^Leu Phe^6.40(272)^Glu Phe^6.40(272)^Lys Phe^6.40(272)^Tyr	Decreased expression Increased expression Undetectable Undetectable Decreased expression	(77) (81)

Ala^6.41(273)^	Met^3.43(132)^–Ala^6.41(273)^	Leu^5.55(228)^–Ala^6.41(273)^			

Asp^7.49(319)^	Phe^6.40(272)^–Asp^7.49(319)^	Met^3.43(132)^–Asp^7.49(319)^	Asp^7.49(318)^Asn (M)^c^ Asp^7.49(318)^Ala (M) Asp^7.49(318)^Glu (M) Asp^7.49(318)^Leu (M)	Decreased coupling efficiency Decreased coupling efficiency Decreased expression Undetectable	(23, 25, 39)

Tyr^7.53(323)^	Phe^1.53(56)^–Tyr^7.53(323)^	Met^3.43(132)^–Tyr^7.53(323)^Ile^3.46(135)^–Tyr^7.53(323)^	Tyr^7.53(323)^Ala Tyr^7.53(322)^Phe (M) Tyr^7.53(323)^Cys	Uncoupled Increased coupling efficiency Decreased coupling efficiency	(36, 39, 77)

Gly^7.54(324)^		Leu^2.43(80)^–Gly^7.54(324)^			

*GnRH receptor residues equivalent to those that form inactive or active conformation-specific interhelical contacts in class A GPCR structures (17, 19) are listed with summaries of the effects of mutagenesis*.

*^a^Undetectable indicates no measurable function mostly due to lack of expression*.

*^b^Rescued by pharmacoperone indicates that pharmacoperone pretreatment of cells increased ligand binding or cellular signaling*.

*^c^Mutations in mouse GnRH receptors are indicated by (M)*.

#### Interactions That Stabilize the Closed G Protein-Binding Pocket

The inactive rhodopsin structure showed a salt bridge between Arg^3.50^ of the D/ERY motif at the cytoplasmic end of TM3, and Glu^6.30^ at the cytosolic end of TM6 (28, 82). This “ionic-lock” interaction stabilizes the inactive GPCR conformation by drawing the cytoplasmic ends of TM3 and TM6 together (26, 83, 84). However, the salt bridge between Arg^3.50^ and Glu^6.30^ cannot provide a universal mechanism for stabilizing inactive GPCR conformations, because Glu^6.30^ is not conserved (83). The GnRH receptor has Arg^6.30(262)^, which clearly cannot form a salt bridge with Arg^3.50(139)^. Nevertheless, mutations show that both Arg residues are important for GnRH receptor structure and function. The cHH-associated Arg^6.30(262)^Gln mutation decreased ligand binding and cellular signaling (85), which was recovered when cells were treated with pharmacoperone (1, 47), suggesting that the Arg^6.30(262)^ side chain forms an intramolecular interaction that stabilizes folding of the unoccupied GnRH receptor. Since GPCRs are biosynthesized in cellular compartments that are inaccessible to endogenous ligands, receptors are likely synthesized in inactive conformations. Inactive conformation-specific interhelical contacts may thus stabilize receptor biosynthesis, so mutations that disrupt these contacts may also disrupt expression.

Mutation of Arg^3.50(139)^ in the DRY/S motif to other basic amino acids, His or Lys, resulted in no measurable receptor function, consistent with disruption of a structurally important interaction. An Arg^3.50(139)^Gln mutation increased GnRH receptor expression and decreased activation of cellular signaling (29). This suggests that the Gln side chain mimics an interhelical interaction of Arg^3.50(139)^ that stabilizes receptor folding and stabilizes the inactive receptor conformation. The importance of the Arg^3.50(139)^ side chain for GnRH receptor structure is also supported by cHH-associated Arg^3.50(139)^His and Arg^3.50(139)^Cys mutations, which are poorly expressed (30, 78), but rescued by pharmacoperone treatment (Table 2) suggesting that the mutations disrupt biosynthesis of the unoccupied (inactive) GnRH receptor. Since Arg^3.50(139)^ cannot form an ionic lock in the GnRH receptor, it must form a different contact. GPCR structures show a conserved inactive conformation-specific interhelical contact of Arg^3.50^ with the 6.37 locus (19). Thus, the Arg^3.50(139)^ side chain may stabilize the inactive GnRH receptor conformation by forming an interhelical hydrogen bond with Thr^6.37(269)^ (Table 2), which may be enhanced in the Arg^3.50(139)^Gln mutant receptor.

Many conserved inactive conformation-specific interhelical contacts involve residues in TM3 and TM6 (19), suggesting that, like the ionic lock, they maintain close proximity of the cytoplasmic ends of TM3 and TM6. Met^3.43(132)^, conserved as a large hydrophobic residue in most GPCRs, is likely to stabilize the inactive GnRH receptor conformation by interacting with Phe^6.40(272)^ and Ala^6.41(273)^ in TM6 (Table 2). Mutation of Met^3.43(132)^ and four mutations of Phe^6.40(272)^ decreased GnRH receptor expression, suggesting that they disrupt receptor biogenesis. In contrast, the Phe^6.40(272)^Leu mutation increased receptor expression (Table 2; Figure S3 in Supplementary Material) (77, 81), suggesting that the Phe^6.40(272)^Leu mutation enhances the TM3-TM6 interhelical contact with Met^3.43(132)^ and so enhances expression of the inactive GnRH receptor.

A more recent study, using five pairs of GPCR structures found that only one TM3–TM6 inactive conformation-specific contact is conserved (17). This interaction between the 3.46 and 6.37 loci is the key contact that defines the “closed” conformation of the G protein-binding site and prevents G protein binding (17). In the GnRH receptor, these residues are Ile^3.46(135)^ and Thr^6.37(269)^ (Table 2). Mutation of Ile^3.46(135)^ to Ala and Val both completely ablated GnRH receptor function (29). In the context of the inactive GPCR crystal structures, the Ala and Val side chains are likely too short to “fulfill the distance criteria for contact formation” (17). Substitution of Ile^3.46(135)^ with Leu, which has a large branched aliphatic side chain, partially preserved GnRH receptor expression and increased coupling efficiency (29). Thus, the Ile^3.46(135)^Leu mutation may destabilize the inactive conformation of the GnRH receptor by weakening the Ile^3.46(135)^–Thr^6.37(269)^ interhelical contact and favoring formation of the R* active conformation. A cHH-associated Thr^6.37(269)^Met mutation resulted in no measurable function in several assay systems (80). This result confirms the importance of Thr^6.37(269)^ in GnRH receptor structure, but provides no information about its function in receptor conformation.

The second key inactive conformation-specific interhelical contact orients Tyr^7.53^ of the NPxxY motif in TM7 toward TM1, where it contacts the residue in the 1.53 locus, keeping the Tyr^7.53^ side chain away from the interior of the TM domain (17, 19). The corresponding residues of the GnRH receptor are Phe^1.53(56)^ in TM1 and Tyr^7.53(323)^ in the N/DPxxY motif in TM7 (Table 2).

#### Conserved Water-Mediated Hydrogen Bond Networks and the Sodium Ion-Binding Pocket

Higher resolution GPCR structures revealed internal water molecules that form a conserved network, with hydrogen bonds connecting the conserved amino acids in different helices, including Asn^1.50^, Asp^2.50^, Trp^6.48^ of the CWxPY motif and residues of the NPxxY motif. The water molecules both stabilize the GPCR structural fold and mediate transition between conformational states, by forming many “low energy switches,” which can be broken and reconfigured during GPCR activation (22, 26, 33, 50, 86). The GnRH receptor likely has a network of intramolecular water molecules between its highly conserved amino acids and mutations that disrupt GnRH receptor expression or function, may do so by disrupting the water-mediated intramolecular network.

The hydrophilic network in inactive GPCRs also includes a sodium ion, which makes a conserved contact with Asp^2.50^ and stabilizes the inactive receptor conformation. Cations decrease GnRH receptor agonist binding (87). Since type 1 GnRH receptors do not have Asp^2.50^, the nearby Glu^2.53(90)^ and Asp^7.49(319)^ (of the N/DPxxY motif) residues may provide negative charges that enhance cation binding, as suggested for the PAR1 receptor (22, 24, 88, 89).

#### The Hydrophobic Barrier and Transmission Switch

All inactive class A GPCR structures have a layer of hydrophobic amino acids on the cytosolic side of Asp^2.50^ that separates the water molecules in the ligand- and G protein-binding pockets. The barrier stabilizes the inactive GPCR conformation and consists of conserved class A GPCR residues, including some that also form conserved inactive conformation-specific interhelical contacts (26, 33, 75). The partially overlapping “central hydrophobic core,” consisting of the conserved Phe^6.44^ and conserved hydrophobic residues in the 3.43 and 6.40 loci (90), the “core triad” consisting of Phe^6.44^, Pro^5.50^ and the hydrophobic residue at position 3.40 (22) and the “transmission switch,” consisting of the hydrophobic residue at position 3.40, Pro^5.50^, Leu^5.51^, and Phe ^6.44^, which is one helical turn away from Trp^6.48^ of CWxPY motif, all couple the ligand-binding pocket to the hydrophobic barrier (26, 31, 32). The GnRH receptor has hydrophobic residues in the loci associated with the hydrophobic barrier and the transmission switch, so it is likely that the GnRH receptor has a hydrophobic barrier that stabilizes its inactive conformation.

### The Active Receptor Structure

The diverse GPCR ligands trigger a variety of receptor-specific molecular changes to initiate receptor activation (31, 91). The changes must converge to generate a structurally conserved G protein-binding pocket that can be recognized by- and activate a G protein that interacts with many GPCRs (17, 18). Structural features that define the activated GPCR conformation include rotation of TM6, changed interfaces of TM3, TM5, and TM6, opening of the hydrophobic barrier, movement of the Tyr^5.58^ (Asn^5.58(231)^ in type 1 GnRH receptors) and Tyr^7.53^ side chains toward the interior of the TM bundle and opening of a cytoplasmic surface cleft that allows G protein access and binding (20, 31, 32, 56, 90, 91).

#### Rotation of TM6 and Activation of the Transmission Switch

In spite of the diversity of ligand-binding surfaces many ligands contact highly conserved residues, particularly Trp^6.48^ and Tyr^6.51^ of the CWxPY motif. In the active structure of rhodopsin retinal isomerization induces movement of Trp^6.48^, which causes rotation of TM6, without changing either the rotamer angle of the Trp^6.48^ side chain or the hinge angle of the proline kink. The exaggerated bend angle of Pro^6.50^ amplifies movement of the cytoplasmic end of TM6, which moves outward, away from TM3 (33). Similar rotation of the cytoplasmic end of TM6 was seen in all fully active GPCR structures (22, 27, 56, 92, 93).

Rotation of TM6 also changes its interhelical contacts with TM3 and TM5. The Phe^6.44^ side chain moves toward TM5, where it rearranges the Leu^5.51^ and Pro^5.50^ residues and moves the conserved Leu^3.40^ of TM3 away from TM5, thus triggering the “transmission switch” (26, 31). Similar activation of the transmission switch *via* agonist-induced movement of Trp^6.48^ is seen in the A_2A_-adenosine and μ-opioid receptors (22, 31). The transmission switch changes the conformation of the proline kink of TM5 and rotates the conserved Tyr^5.58^ side chain (near the cytoplasmic end) inwards. It also changes the position of TM3, rotating it and moving it slightly toward the extracellular of side the TM domain (90).

Active structures of other GPCRs showed similar outward movement of the cytosolic end of TM6 but showed no agonist contact with Trp^6.48^ (31, 93–98), so TM6 movement must be achieved *via* different mechanisms. In the β_2_-adrenergic receptor, agonist binding at the extracellular end of TM5 causes movement of Pro^5.50^, which moves Ile^3.40^ in TM3 and Phe^6.44^ in TM6, thus opening the core triad, triggering the transmission switch and rotating the cytoplasmic end of TM6 away from the helix bundle (26, 31). A similar opening of the core triad residues occurs in the μ-opioid receptor, except that the agonist binds to two residues (the 3.32 and 3.36 loci) in TM3, resulting in movement of Ile^3.40^, which activates the core triad (22). Mutation of the Lys^3.32(121)^ residue in TM3 of the GnRH receptor decreased binding of GnRH agonists but not antagonists (99), suggesting that GnRH interaction with Lys^3.32(121)^ initiates activation. Although mutagenesis experiments do not support a role for Trp^6.48(280)^ in ligand binding or activation of the GnRH receptor (38), there is evidence that GnRH contacts Tyr^6.58(290)^ at the extracellular end of TM6 (100) and that Tyr^6.51(283)^ of the CWxPY affects ligand-binding affinity (37), suggesting that these residues may initiate rotation of TM6.

#### Reconfiguration of the Water-Mediated Polar Network and Opening of the Hydrophobic Barrier and G Protein-Binding Pocket

The water-mediated polar network in active GPCR structures differs from that of inactive structures. Agonist-induced movement of the extracellular ends of the TM helices rearranges water molecules at the extracellular side of the TM domain and opens the hydrophobic barrier, which allows formation of a continuous water channel between the ligand-binding pocket and the cytoplasmic surface of the receptor. This changes the conformation of TM7 and causes rotation of Tyr^7.53^ away from its interaction with TM1 toward the center of the TM bundle (22, 32, 33, 50, 75, 90, 96, 101, 102). The rearranged water molecules link Arg^3.50^ with Tyr^5.58^ and Tyr^7.53^ in a water-mediated interhelical network that can be considered the “open” conformation of the ionic lock that stabilizes the active receptor conformation (22, 28, 96). Movements of TM6 and TM7 collapse the sodium ion-binding pocket, making it too small to accommodate the ion (24, 88, 97) and the cation moves toward the cytoplasm through the open hydrophobic barrier (24). Mutation of the water-associated Arg^3.50(139)^, Asp^7.49(318)^ and Tyr^7.53(322)^ residues decreases GnRH receptor coupling efficiency (23, 29, 30, 39, 77, 103) indicating that they have roles in the active receptor conformation, which may be mediated by the water network.

Rotation of TM6 and opening of the hydrophobic barrier break the inactive conformation-specific interhelical contacts and form new active conformation-specific interhelical contacts. The ionic lock opens and the Arg^3.50^ side chain moves into the space vacated by TM6 where it orients toward Tyr^5.58^ of TM5. Arg^3.50^ and Tyr^5.58^ form new interhelical contacts with the hydrophobic residue in position 6.40 (19, 22, 28). The GnRH receptor has Asn^5.58(231)^, which is smaller than Tyr. The NTSR1 neurotensin receptor also has Asn^5.58^ and an “active-like” NTSR1 structure shows a hydrogen bond between Asn^5.58(257)^ and Arg^3.50(167)^ (104), which suggests that Asn^5.58(231)^ stabilizes the open ionic lock in the GnRH receptor.

On activation Met^3.43^ breaks its inactive conformation-specific interhelical contact with the residues in positions 6.40 and 6.41. The release of the hydrophobic side chain in position 6.41 allows it to contact the hydrophobic residue in position 5.55, forming one of two key active conformation-specific interhelical contacts (17, 19). In the GnRH receptor these residues are Leu^5.55(228)^ and Ala^6.41(273)^ (Table 2). One helical turn closer to the cytoplasmic side of the receptor, the Ile^3.46^ side chain breaks its inactive conformation-specific contact with the position 6.37 side chain and forms a new interhelical contact with Tyr^7.53^, forming the second key active conformation-specific interaction (17, 19). The breaking of the TM3-TM6 contacts opens a cleft that allows G protein access and releases the position 6.37 residue to make a conserved interaction with the G protein (17, 27) (Figure S3 in Supplementary Material). In the GnRH receptor these residues are Ile^3.46(135)^, Thr^6.37(269)^ and Tyr^7.53(323)^ (Table 2). Mutation of Ile^3.46(135)^ to Leu increased GnRH receptor coupling efficiency (29), suggesting that the Leu side chain may favor interaction with Tyr^7.53(323)^ over interaction with Thr^6.37(269)^, thus favoring the active conformation. A role for Tyr^7.53(323)^ in stabilizing the active GnRH receptor conformation is supported by the Tyr^7.53(323)^Ala mutant, which did not activate cellular signaling (39, 77).

## Ligand-Binding Interactions

Gonadotropin-releasing hormone is a decapeptide with the sequence pGlu^1^-His^2^-Trp^3^-Ser^4^-Tyr^5^-Gly^6^-Leu^7^-Arg^8^-Pro^9^-Gly^10^NH_2_. The amino-terminal residues, pGlu^1^, His^2^, and Trp^3^, determine agonist activity, but the carboxy-terminal residues, particularly Arg^8^, are necessary for high affinity binding to the GnRH receptor (4, 13, 105). Although the GnRH peptide is conformationally flexible, the predominant conformer consists of a β-turn that brings the amino- and carboxy-termini close together. This conformation is stabilized by substituting the achiral Gly^6^ residue of the GnRH peptide with d-amino acids, which increase receptor-binding affinity, whereas l-amino acids decrease affinity (4, 13, 105). In the absence of a GnRH-receptor crystal structure, alanine-scanning mutagenesis and molecular models have identified potential intermolecular contacts. However, most have not been validated by biochemical studies to distinguish indirect disruption of the GnRH-binding surface (Figure S4 in Supplementary Material). We will discuss potential GnRH-receptor contacts in the context of peptide-bound GPCR structures and recent GnRH receptor mutagenesis studies.

### The Consensus Ligand-Binding Pocket in the GnRH Receptor

In spite of the diversity of GPCR ligand-binding pockets, Venkatakrishnan et al. identified a consensus ligand-binding pocket consisting of topologically equivalent residues at positions 3.32, 3.33, and 3.36 in TM3, 6.48 and 6.51 in the CWxPY motif, and 7.39 in TM7. These residues include two conserved interhelical contacts, 3.36–6.48 and 6.51–7.39, which couple the ligand-binding pocket to the conserved GPCR structure (20). Structures of peptide-bound GPCRs show that the sections of the peptides that are required for agonist activity, the carboxy-termini of endothelin-1 and apelin and the amino-termini of chemokines, penetrate the TM cores of their receptors and interact with subsets of the consensus ligand-binding residues, whereas other parts of the peptides bind outside of the core (93, 106–109). This suggests that the amino-terminal residues of GnRH may interact with the consensus-binding pocket.

#### Lys^3.32(121)^

The endothelin-1 peptide penetrates the TM core of the ET_B_-endothelin receptor and contacts the consensus-binding residues, Trp^6.48^ and Leu^6.51^ and Gln^3.32^ (107). The smaller peptide agonist NTS_8–13_ binds closer to the extracellular surface of the NTSR1 neurotensin receptor, but may contact the consensus Arg^3.32(149)^ residue (104). Mutation of the equivalent GnRH receptor residue, Lys^3.32(121)^, to Gln or Ala decreased GnRH affinity and signaling, but had minimal effect on binding of a peptide antagonist, which had modified amino-terminal residues. This led to a conclusion that Lys^3.32(121)^ may form a hydrogen bond with the aromatic rings of His^2^ or Trp^3^ of GnRH (37, 99). Subsequent models proposed that Lys^3.32(121)^ contacts pGlu^1^ or His^2^ (37, 68, 110–113) but, in the absence of further experiments, it remains uncertain whether Lys^3.32(121)^ directly contacts GnRH or initiates receptor activation.

#### Trp^6.48(280)^

Agonist peptide ligands bound to the NTSR1 neurotensin, US28 viral chemokine and apelin receptors do not penetrate deeply enough to contact Trp^6.48^ of the CWxPY motif (93, 104, 106) and mutagenesis of Trp^6.48^ had minimal effects on NTSR1 receptor function (114). Molecular models suggested that Trp^6.48(280)^ of the GnRH receptor interacts with Trp^3^ of the GnRH peptide (13, 69, 74, 111). However, mutations of Trp^6.48(280)^ had minimal effects on GnRH affinity or cellular signaling (37, 38), indicating that it does not directly contact GnRH. Since TM3 has central roles in ligand binding, the conserved interhelical network and the hydrophobic core, movement of TM3 may provide an alternative molecular pathway to Trp^6.48^-mediated activation of the transmission switch in the GnRH and NTSR1 receptors (20, 57).

#### Tyr^6.51(283)^ and Phe^7.39(309)^

The hydrophobic residue in position 6.51 contacts the ligand in the ET_B_-endothelin and apelin receptors (106, 107), whereas the conserved Glu^7.39^ of chemokine receptors is a key determinant of chemokine binding and receptor activation (109). A Tyr^6.51(283)^Phe mutation in the GnRH receptor and mutations of Phe^7.39(309)^ to Leu or Gln decreased GnRH-binding affinity (37). A recent computational model suggests that Phe^7.39(309)^ may contact Trp^3^ of GnRH (110). Together, these data are consistent with the mutations disrupting ligand binding by breaking an interhelical contact between residues that may also contact the ligand, but more experiments are needed.

In summary, it is possible that amino-terminal residues of the GnRH peptide contact some of the consensus ligand-binding residues, Lys^3.32(121)^, Tyr^6.51(283)^, and Phe^7.39(309)^, but not Trp^6.48(280)^. It remains uncertain whether GnRH binds to a largely extracellular surface of the receptor like NTS_8–13_ (104) or penetrates the TM core like the peptide ligands of the apelin and chemokine receptors (106, 109).

### GnRH Interactions Outside of the Consensus Ligand Pocket

#### The Amino Terminus, TM2, and Extracellular Loop 1

A molecular model predicted that Arg^1.35(38)^ in the amino terminus of the GnRH receptor is close to the carboxy-terminal Pro^9^-Gly^10^NH_2_ of GnRH. Mutations of Arg^1.35(38)^ decreased GnRH-binding affinity, but had lesser effects on binding of [Pro^9^-NHEt]-GnRH, which lacks Gly^10^NH_2_ (112). The results support a hydrogen bond contact between Arg^1.35(38)^ and Gly^10^NH_2_, but show that both the geometry and charge of the Arg^1.35(38)^ side chain are important for additional inter- or intramolecular interactions. Asn^2.65(102)^ in TM2 has similar functions in distinguishing Gly^10^NH_2_ of GnRH (13, 115). These studies suggest that the carboxy-terminus of GnRH may locate close to both Arg^1.35(38)^ and Asn^2.65(102)^ (Figure S4 in Supplementary Material). Systematic mutagenesis of Asp^2.61(98)^ of the GnRH receptor, combined with ligand modification, showed that the Asp^2.61(98)^ side chain determines receptor recognition of His^2^ of the GnRH peptide, *via* a hydrogen bond, whereas the charge of the Asp^2.61(98)^ side chain may configure the surface of the ligand-binding pocket by forming an interhelical salt bridge with Lys^3.32(121)^ (13, 116). Thus, residues in the amino-terminus and extracellular ends of TM1 and TM2 of the GnRH receptor appear to affect GnRH binding *via* direct contacts with amino- and carboxy-terminal residues of the peptide and *via* intramolecular interactions that affect the shape of the ligand-binding surface.

#### TM6, Extracellular Loop 3, and TM7

Molecular models of the GnRH receptor showed contact of Tyr^6.58(290)^, two helical turns toward the extracellular end of TM6 from the CWxPY motif, with the Tyr^5^ side chain of the GnRH peptide. Systematic mutagenesis showed that both the hydroxyl group and the aromatic ring of the Tyr^6.58(290)^ side chain contribute to high affinity binding of GnRH, but had less effect on binding of [Ala^5^]-GnRH, consistent with the hydroxyl group of Tyr^6.58(290)^ interacting with the aromatic ring of Tyr^5^ of the peptide. The receptor mutations also decreased GnRH potency in signaling assays more than they decreased binding affinity, showing that the Tyr^6.58(290)^ side chain has an additional role in coupling agonist binding to receptor activation (100). The Tyr^6.58(290)^–Tyr^5^ interaction may initiate movement and rotation of TM6 in the GnRH receptor.

Mutation of His^7.36(305)^ at the extracellular end of TM7 in the mouse GnRH receptor to non-polar amino acids decreased GnRH-binding affinity, suggesting loss of a hydrogen bond interaction. Ligand modification suggested a His^7.36(305)^-Trp^3^ hydrogen bond contact. However, mutation of His^7.36(305)^ to polar amino acids had no effect on ligand-binding affinity, making a direct interaction with the ligand unlikely. Molecular modeling showed that His^7.36(305)^ made only intramolecular contacts with the amino terminus of the receptor, whereas Trp^3^ of GnRH was oriented near the consensus ligand-binding residue, Phe^7.39(308)^. This suggests that His^7.36(305)^ forms an interhelical contact that positions Phe^7.39(308)^ to form π–π contact with Trp^3^ of the peptide (110). The cHH-associated GnRH receptor mutation, Thr^32^Ile, which decreases ligand-binding affinity (63), is immediately adjacent to the His^7.36(305)^ interhelical contacts and may disrupt the interhelical contact.

Mammalian GnRH has a basic Arg^8^ residue, which is important for binding to type 1 GnRH receptors. Mutation of the acidic Asp^7.32(302)^ residue to uncharged Asn decreased binding affinity of GnRH, but had no effect on binding of peptides with uncharged Gln^8^. This suggests that Asp^7.32(302)^ forms a salt bridge contact with Arg^8^ of the peptide. However, “conformationally constrained” GnRH peptides, in which the high affinity β-turn was stabilized by a d-amino acid in position 6, retained high affinity binding in the absence of Asp^7.32(302)^ or Arg^8^ or both. Since the Arg^8^ side chain also contributes to stabilizing the β-turn in the native GnRH peptide, it was concluded that the interaction of Asp^7.32(302)^ with Arg^8^ induces the high-affinity peptide conformation (70, 117).

### Conformationally Constrained GnRH Peptides

Although it was hypothesized that the Asp^7.32(302)^-Arg^8^ interaction induced the high affinity conformation of GnRH on binding to the receptor, mutation of many different GnRH receptor residues causes a similar large decrease in binding affinity of native GnRH, but much smaller decreases in affinity for conformationally constrained GnRH peptides (37, 68, 100, 115). So the ability of constrained peptides to overcome the ligand-binding affinity effects of receptor mutations is not specific to the Asp^7.32(302)^-Arg^8^ interaction. In the active GPCR conformations that have increased agonist-binding affinity, a “cap” forms over the extracellular surface of the ligand-binding pocket and increases agonist affinity by hindering dissociation of the ligand and trapping it in the binding pocket (51). In peptide receptors the larger ligand extends beyond the TM-binding pocket, so it cannot be capped. Nevertheless, the extracellular sides of agonist-bound peptide receptors, such as the NTSR1 neurotensin and μ-opioid receptors, move inwards and it has been suggested that this movement “pinches” the peptide ligand, increasing its affinity by hindering its dissociation (51). Comparison of the ET_B_-endothelin receptor structures with and without endothelin-1 showed that the peptide induces inward movement of the extracellular ends of the TM helices “tightening” the ligand pocket (107). Extrapolating to the GnRH receptor, conformationally constrained peptides may be more compact than GnRH before contacting the receptor and hence enhance narrowing of the ligand-binding pocket *via* multiple contacts with the ligand-binding pocket. The tightening would overcome mutation-induced loss of individual contacts.

## Concluding Remarks

Although only direct determination will confirm the GnRH receptor structure, growing numbers of other GPCR structures provide insight into common features likely to be shared by the GnRH receptor. GPCR structures can be used to hypothesize mechanisms by which agonist binding is coupled to G protein activation, which must be tested by dynamic methods, such as site-directed mutagenesis and functional analysis, regardless of availability of directly determined structures. Identification of the conformation-independent interhelical contact network has provided explanations for decreased expression of many cHH-associated mutant GnRH receptors, whereas the conserved conformation-specific interhelical contacts begin to explain how conserved residues mediate receptor activation. In spite of the diversity of ligand-binding surfaces, recent agonist-bound peptide-binding GPCRs suggest that ligand contacts in TM3 may trigger receptor activation and they may explain the high affinity of conformationally constrained GnRH peptides.

## Author Contributions

CF conceived the project and wrote the manuscript. AM wrote a preliminary review as part of her MSc dissertation, which was partly used in the current project.

## Conflict of Interest Statement

The authors declare that the research was conducted in the absence of any commercial or financial relationships that could be construed as a potential conflict of interest.
