# Supplementary material for: Gonadotropin-Releasing Hormone (GnRH) Receptor Structure and GnRH Binding
Source: Front Endocrinol (Lausanne). 2017 Oct 24;8:274. doi: 10.3389/fendo.2017.00274 (PMC5662886; doi:10.3389/fendo.2017.00274)
Supplement: Supplementary file 1 [file Table_1.PDF]

Supplementary Material

GnRH Receptor Structure and GnRH Binding

Colleen A. Flanagan*, Ashmeetha Manilall

*** Correspondence:** Dr. Colleen A Flanagan: Colleen.Flanagan@wits.ac.za

1 Supplementary Figures and Tables

1.1 Supplementary Tables

Table S1. GnRH receptor residues potentially involved in conserved conformation-independent interhelical residue contacts. GnRH receptor residues in the loci that form conserved interhelical contacts in both active and inactive GPCR structures as identified by (1) and (2) are listed with the expected interhelical contacts. To assess the relevance of these interactions in GnRH receptor structure and function, the effects of mutations are listed.

GnRH receptor residue	Predicted contacts based on conserved interhelical contacts	GnRH receptor mutations	Effects of mutations	References
Phe$^{1.43(46)}$	F$^{1.43}$-T$^{2.54}$, F$^{1.43}$-M$^{2.58}$	-	-	
Ser$^{1.46(49)}$	S$^{1.46}$-T$^{2.54}$, S$^{1.46}$-C$^{7.47}$	-	-	
Phe$^{1.49(52)}$	F$^{1.49}$-P$^{7.50}$	-	-	
Asn$^{1.50(53)}$	N$^{1.50}$-T$^{2.47}$, N$^{1.50}$-N$^{2.50}$, N$^{1.50}$-L$^{2.51}$, N$^{1.50}$-P$^{7.46}$, N$^{1.50}$-P$^{7.50}$	Asn$^{1.50(53)}$Ala (M)[a]<br>Asn$^{1.50(53)}$Asp (M)	undetectable[b]<br>undetectable	(3)
Phe$^{1.53(56)}$	F$^{1.53}$-T$^{2.47}$	-	-	
Leu$^{1.54(57)}$	L$^{1.54}$-T$^{2.47}$	-	-	
Leu$^{1.57(60)}$	L$^{1.57}$-K$^{2.44}$	-	-	

$Leu^{2.42(79)}$	$L^{2.42}$-$V^{3.45}$, $L^{2.42}$-$I^{3.46}$	-	-	
$Leu^{2.43(80)}$	$L^{2.43}$-$Y^{7.53}$	$Leu^{2.43(80)}$Ala	decreased expression and decreased agonist potency	(4)
$Lys^{2.44(81)}$	$L^{1.57}$-$K^{2.44}$	-	-	
$His^{2.45(82)}$	$H^{2.45}$-$M^{3.43}$, $H^{2.45}$-$V^{4.46}$	-	-	
$Leu^{2.46(83)}$	$L^{2.46}$-$P^{3.39}$, $L^{2.46}$-$M^{3.42}$	-	-	
$Thr^{2.47(84)}$	$N^{1.50}$-$T^{2.47}$, $F^{1.53}$-$T^{2.47}$, $L^{1.54}$-$T^{2.47}$	-	-	
$Asn^{2.50(87)}$	$N^{1.50}$-$D^{2.50}$, $N^{2.50}$-$P^{7.46}$	$Asn^{2.50(87)}$Ala<br>$Asn^{2.50(87)}$Asp (M)<br>$Asn^{2.50(87)}$Gln (M)	undetectable	(3, 5-7)
$Leu^{2.51(88)}$	$N^{1.50}$-$L^{2.51}$	-	-	
$Glu^{2.53(90)}$	$E^{2.53}$-$S^{3.35}$	$Glu^{2.53(90)}$Ala<br>$Glu^{2.53(90)}$Gln (M)<br>$Glu^{2.53(90)}$Lys<br>$Glu^{2.53(90)}$Asp	undetectable<br>normal function<br>cHH, undetectable, rescued by pharmacoperone[c]<br>cHH-associated mutation	(7)<br>(8)<br>(9-11)<br>(12)
$Thr^{2.54(91)}$	$F^{1.43}$-$T^{2.54}$	-	-	
$Met^{2.58(95)}$	$F^{1.43}$-$M^{2.58}$	-	-	
$Phe^{3.34(123)}$	$F^{3.34}$-$S^{4.53}$, $F^{3.34}$-$A^{4.57}$	-	-	
$Ser^{3.35(124)}$	$E^{2.53}$-$S^{3.35}$	$Ser^{3.35(124)}$Ala<br>$Ser^{3.35(124)}$Asp	 undetectable	(13)
$Met^{3.36(125)}$	$M^{3.36}$-$W^{6.48}$	-	-	
$Tyr^{3.37(126)}$	$Y^{3.37}$-$S^{4.53}$	-	-	
$Ala^{3.38(127)}$	$A^{3.38}$-$W^{4.50}$, $A^{3.38}$-$S^{4.53}$	-	-	
$Pro^{3.39(128)}$	$L^{2.46}$-$P^{3.39}$	-	-	
$Ala^{3.40(129)}$	$A^{3.40}$-$F^{6.44}$	$Ala^{3.40(129)}$Asp	cHH, undetectable, rescued by pharmacoperone	(10, 14, 15)
$Phe^{3.41(130)}$	$F^{3.41}$-$A^{4.49}$	-	-	

$Met^{3.42(131)}$	$L^{2.46}$-$M^{3.42}$	-	-	
$Met^{3.43(132)}$	$H^{2.45}$-$M^{3.43}$, $M^{3.43}$-$F^{6.44}$	$Met^{3.43(132)}Ala$	undetectable, partially rescued by pharmacoperone	(16)
$Val^{3.44(133)}$	$V^{3.44}$-$M^{5.54}$	-	-	
$Val^{3.45(134)}$	$L^{2.42}$-$V^{3.45}$	-	-	
$Ile^{3.46(135)}$	$L^{2.42}$-$I^{3.46}$	$Ile^{3.46(135)}Ala$<br>$Ile^{3.46(135)}Leu$<br>$Ile^{3.46(135)}Val$	undetectable<br>decreased expression<br>undetectable	(17)
$Ser^{3.47(136)}$	$S^{3.47}$-$C^{5.57}$	-	-	
$Ser^{3.51(140)}$	$S^{3.51}$-$C^{5.57}$, $S^{3.51}$-$K^{5.60}$, $S^{3.51}$-$I^{5.61}$	$Ser^{3.51(140)}Ala$ (M)<br>$Ser^{3.51(140)}Tyr$ (M)	no effect<br>no effect on expression, increased affinity	(18, 19)
$Val^{4.46(160)}$	$H^{2.45}$-$V^{4.46}$	-	-	
$Ala^{4.49(163)}$	$F^{3.41}$-$A^{4.49}$	-	-	
$Trp^{4.50(164)}$	$A^{3.38}$-$W^{4.50}$	-	-	
$Ser^{4.53(167)}$	$F^{3.34}$-$S^{4.53}$, $Y^{3.37}$-$S^{4.53}$, $A^{3.38}$-$S^{4.53}$	-	-	
$Ala^{4.57(171)}$	$F^{3.34}$-$A^{4.57}$	$Ala^{4.57(171)}Thr$	cHH, undetectable, pharmacoperone effect not reported	(20, 21)
$Phe^{5.47(220)}$	$F^{5.47}$-$Y^{6.52}$	$Phe^{5.47(220)}Ala$	undetectable	(16)
$Met^{5.54(227)}$	$V^{3.44}$-$M^{5.54}$, $M^{5.54}$-$A^{6.41}$	$Met^{5.54(227)}Ala$	undetectable	(16)
$Cys^{5.57(230)}$	$S^{3.47}$-$C^{5.57}$, $S^{3.51}$-$C^{5.57}$	-	-	
$Lys^{5.60(233)}$	$S^{3.51}$-$K^{5.60}$	-	-	
$Ile^{5.61(234)}$	$S^{3.51}$-$I^{5.61}$	-	-	
$Ala^{6.41(273)}$	$M^{5.54}$-$A^{6.41}$	-	-	
$Phe^{6.44(276)}$	$A^{3.40}$-$F^{6.44}$, $M^{3.43}$-$F^{6.44}$	$Phe^{6.44(276)}Leu$<br>$Phe^{6.44(276)}Tyr$<br>$Phe^{6.44(276)}Ala$	undetectable<br>decreased binding<br>undetectable	(22)<br><br>(16)

Cys$^{6.47(279)}$	C$^{6.47}$-F$^{7.41}$, C$^{6.47}$-A$^{7.42}$, C$^{6.47}$-N$^{7.45}$	Cys$^{6.47(279)}$Tyr<br>Cys$^{6.47(279)}$Ala	cHH, very low signaling, rescued by pharmacoperone	(10, 11, 21, 23, 24)
Trp$^{6.48(280)}$	M$^{3.36}$-W$^{6.48}$, W$^{6.48}$-A$^{7.42}$	Trp$^{6.48(280)}$Ala<br>Trp$^{6.48(280)}$His<br>Trp$^{6.48(280)}$Ser<br>Trp$^{6.48(280)}$Gln<br>Trp$^{6.48(280)}$Met	decreased expression, rescued by pharmacoperone, no effect on affinity or coupling efficiency.	(25)
		Trp$^{6.48(279)}$Arg (R)<br>Trp$^{6.48(279)}$Ser (R)	undetectable,<br>undetectable	(26)
Tyr$^{6.51(283)}$	Y$^{6.51}$-F$^{7.38}$, Y$^{6.51}$-F$^{7.39}$	Tyr$^{6.51(283)}$Ala	undetectable	(27)
		Tyr$^{6.51(283)}$Phe<br>Tyr$^{6.51(283)}$Leu	decreased binding affinity<br>undetectable	(13)
		Tyr$^{6.51(283)}$His	cHH, undetectable, pharmacoperone not reported	(28, 29)
Tyr$^{6.52(284)}$	F$^{5.47}$-Y$^{6.52}$	Tyr$^{6.52(284)}$Ala	undetectable	(27)
		Tyr$^{6.52(284)}$Leu	decreased binding affinity	(13)
		Tyr$^{6.52(284)}$Cys	cHH, decreased expression, rescued by pharmacoperone	(10, 30)
Phe$^{7.38(308)}$	Y$^{6.51}$-F$^{7.38}$	-	-	
Phe$^{7.39(309)}$	Y$^{6.51}$-F$^{7.39}$	Phe$^{7.39(309)}$Leu<br>Phe$^{7.39(309)}$Gln	decreased binding affinity<br>decreased binding affinity	(13)
Phe$^{7.41(311)}$	C$^{6.47}$-F$^{7.41}$	-	-	
Ala$^{7.42(312)}$	C$^{6.47}$-A$^{7.42}$, W$^{6.48}$-A$^{7.42}$	-	-	
Asn$^{7.45(315)}$	C$^{6.47}$-N$^{7.45}$	Asn$^{7.45(315)}$Ala	undetectable, rescued by pharmacoperone	(24)
Pro$^{7.46(316)}$	N$^{2.50}$-P$^{7.46}$	-	-	
Cys$^{7.47(317)}$	S$^{1.46}$-C$^{7.47}$	-	-	
Pro$^{7.50(320)}$	F$^{1.49}$-P$^{7.50}$, N$^{1.50}$-P$^{7.50}$	Pro$^{7.50(320)}$Leu<br>Pro$^{7.50(320)}$Arg	cHH, undetectable, not rescued by pharmacoperone	(31, 32)

$Tyr^{7.53(323)}$	$L^{2.43}$-$Y^{7.53}$	$Tyr^{7.53(323)}Ala$	increased expression	(6, 16)
		$Tyr^{7.53(322)}Phe$ (M)	decreased expression	(31)
		$Tyr^{7.53(323)}Cys$	cHH, decreased expression, rescued by pharmacoperone	

[a]Mutations in GnRH receptors from species other than human are indicated by (M) for mouse, (R) for rat.

[b]undetectable indicates that no function could be measured and it is expected that receptor structure was disrupted enough to result in transport of undetectable amounts of mutant GnRH receptor protein to the plasma membrane.

[c]rescued by pharmacoperone indicates that pharmacoperone pretreatment of cells transfected with mutant receptor increased the amount of cellular signal production and/or ligand binding, indicating increased expression of the mutant GnRH receptor in the plasma membrane.

1.2 Supplementary Figures

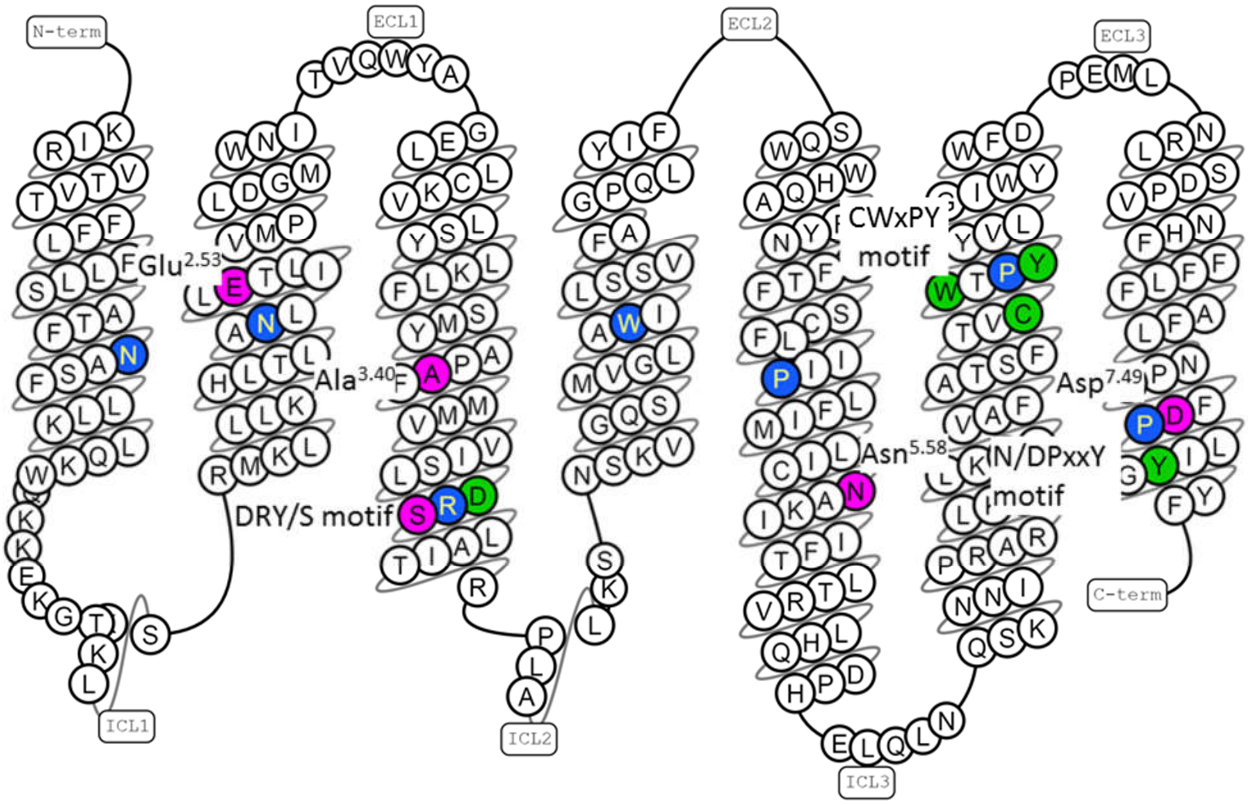

Figure S1. Primary structure of the TM domains of the human GnRH receptor. The snake diagram was downloaded from GPCRdb (www.gpcrdb.org/structure/homology_models) (33). Reference residues of the Ballesteros and Weinstein numbering system are shown in blue, other Class A GPCR-conserved residues are in green and conserved residues that differ in the GnRH receptor are pink. The latter include Glu$^{2.53(90)}$, which is usually a large hydrophobic, Ala$^{3.40(129)}$, which is usually a large hydrophobic, Ser$^{3.51(140)}$, which is usually Tyr in the DRY/S motif, Asn$^{5.58(231)}$, which is usually Tyr, and Asp$^{7.49(319)}$ of the N/DPxxY motif.

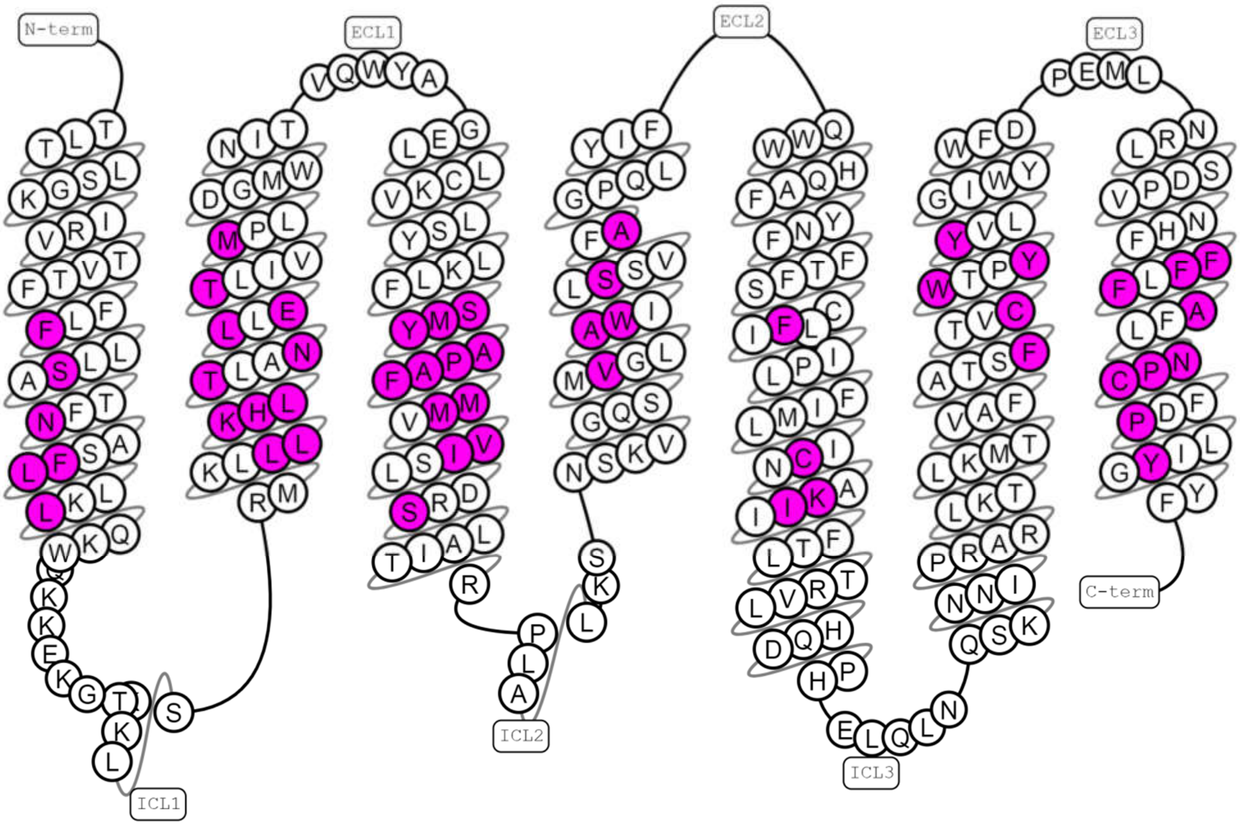

Figure S2. Human GnRH receptor residues topologically equivalent to GPCR residues that form conserved conformation-independent interhelical contacts. The snake diagram was downloaded from GPCRdb (www.gpcrdb.org/structure/homology_models) (33). The GnRH receptor residues topologically equivalent to residues that form conserved interhelical contacts in both active and inactive GPCR structures as identified by (1) and (2) and listed with their conserved interactions in Supplementary Table 1 are shown in pink.

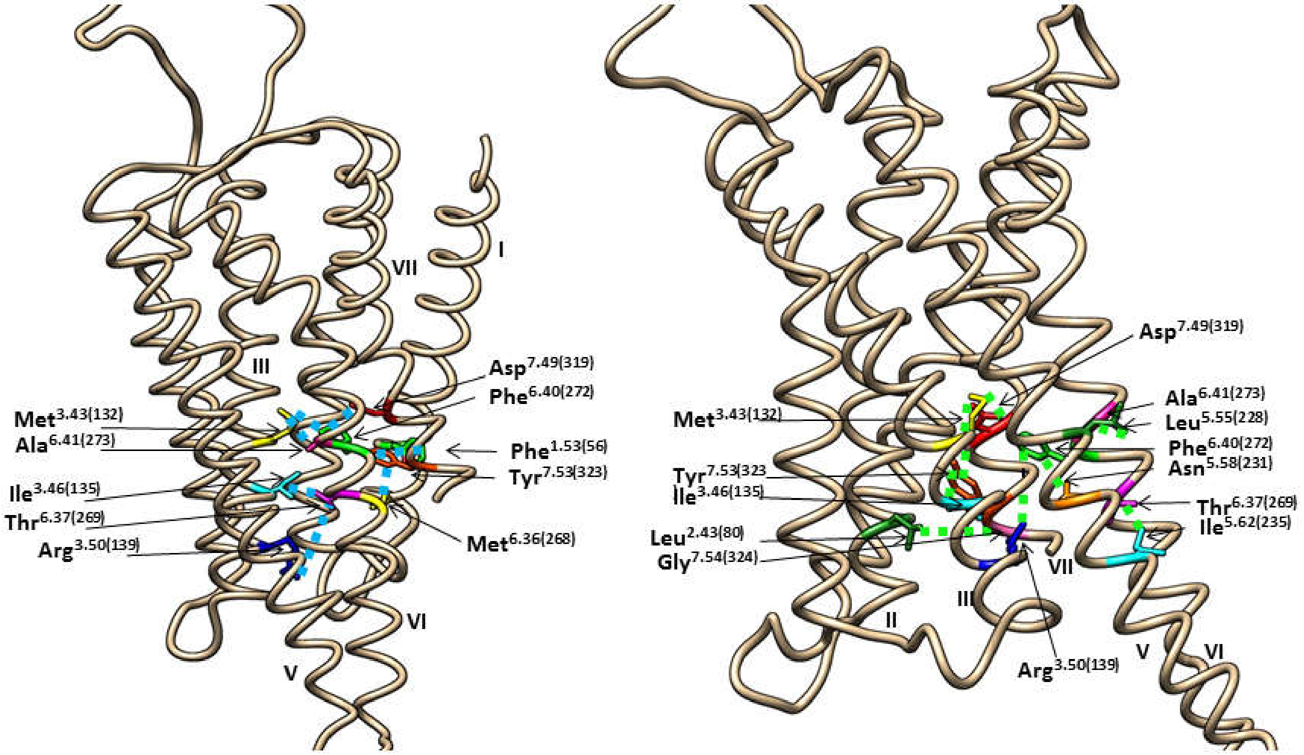

Figure S3. Locations of topologically conserved conformation-specific interhelical contacts in the GnRH receptor. Homology models of the inactive (left) and active (right) conformations of the human GnRH receptor were downloaded from GPCRdb (www.gpcrdb.org/structure/homology_models) (33) and viewed using the UCSF Chimera software package (34). The GnRH receptor residues topologically equivalent to residues that form conserved inactive conformation-specific interhelical contacts as identified by (1) and (2) are shown on the inactive conformation (left) and potential interhelical contacts are indicated by blue dotted lines. Residues topologically equivalent to residues that form conserved active conformation-specific interhelical contacts as identified by (1) and (2) are shown on the active conformation (right) and potential interhelical contacts are indicated by green dotted lines. TM helices are identified by roman numerals.

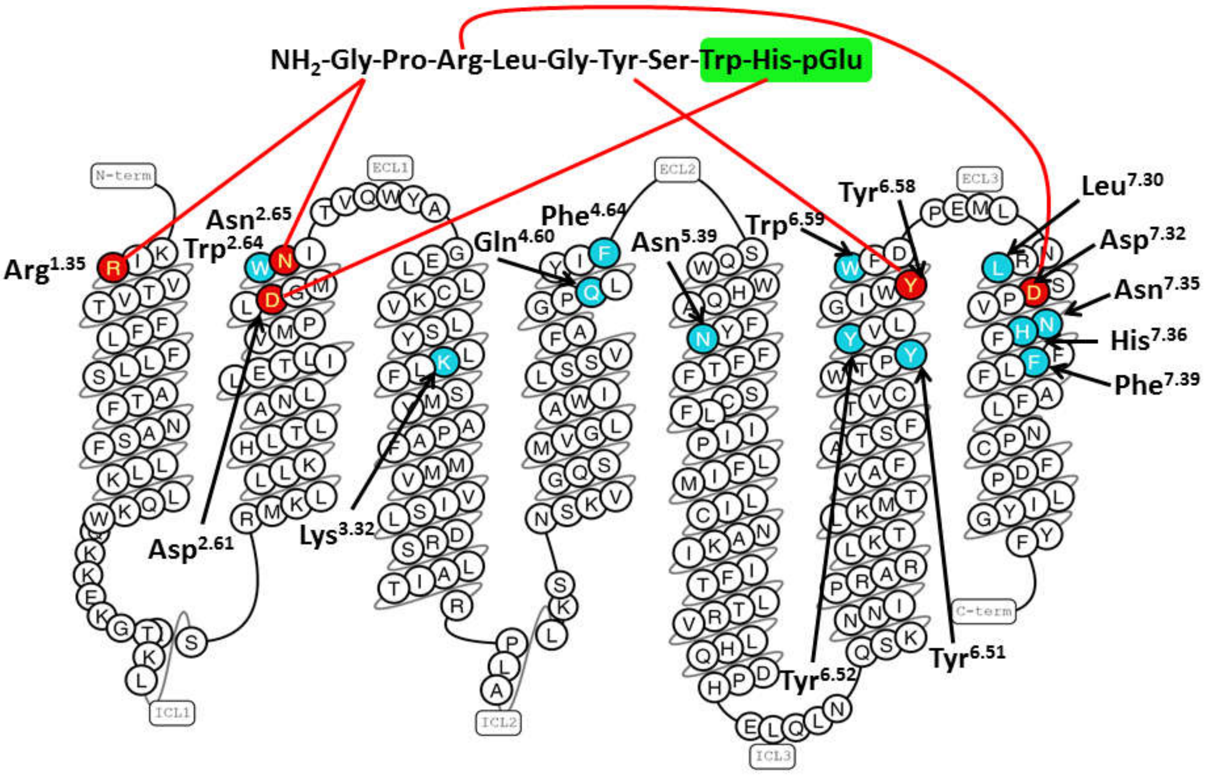

Figure S4. GnRH receptor residues important for binding GnRH. The sequence of the GnRH peptide is shown from the carboxy-terminal Gly-NH_2 on the left, to the amino-terminal pGlu on the right, with the amino acids that are most important for agonist activity highlighted in green. The human GnRH receptor snake diagram was downloaded from GPCRdb (www.gpcrdb.org/structure/homology_models) (33). Receptor residues are colored blue if site-directed mutagenesis of them has been shown to decrease GnRH binding affinity or signaling and they are included as part of the GnRH or GnRH peptide agonist binding surface in one or more molecular models of GnRH receptor ligand binding (13, 27, 35-39). Receptor residues are colored red if multiple mutations and ligand modifications support contacts with specific residues of the GnRH peptide, as indicated by the red connecting lines (8, 40-44).

2 References

1. Cvicek V, Goddard WA, 3rd, Abrol R. Structure-Based Sequence Alignment of the Transmembrane Domains of All Human GPCRs: Phylogenetic, Structural and Functional Implications. *PLoS Comput Biol* (2016) **12**(3):e1004805. doi: 10.1371/journal.pcbi.1004805. PubMed PMID: 27028541; PubMed Central PMCID: PMC4814114.

2. Venkatakrishnan AJ, Deupi X, Lebon G, Tate CG, Schertler GF, Babu MM. Molecular signatures of G-protein-coupled receptors. *Nature* (2013) **494**(7436):185-94. doi: 10.1038/nature11896. PubMed PMID: 23407534.

3. Flanagan CA, Zhou W, Chi L, Yuen T, Rodic V, Robertson D, et al. The functional microdomain in transmembrane helices 2 and 7 regulates expression, activation, and coupling pathways of the gonadotropin- releasing hormone receptor. *J Biol Chem* (1999) **274**(41):28880-6.

4. Arora KK, Krsmanovic LZ, Mores N, O'Farrell H, Catt KJ. Mediation of cyclic AMP signaling by the first intracellular loop of the gonadotropin-releasing hormone receptor. *J Biol Chem* (1998) **273**(40):25581-6.

5. Zhou W, Flanagan C, Ballesteros JA, Konvicka K, Davidson JS, Weinstein H, et al. A reciprocal mutation supports helix 2 and helix 7 proximity in the gonadotropin-releasing hormone receptor. *Mol Pharmacol* (1994) **45**(2):165-70.

6. Arora KK, Cheng Z, Catt KJ. Dependence of agonist activation on an aromatic moiety in the DPLIY motif of the gonadotropin-releasing hormone receptor. *Mol Endocrinol* (1996) **10**(8):979-86.

7. Hoffmann SH, ter Laak T, Kuhne R, Reilander H, Beckers T. Residues within transmembrane helices 2 and 5 of the human gonadotropin- releasing hormone receptor contribute to agonist and antagonist binding [In Process Citation]. *Mol Endocrinol* (2000) **14**(7):1099-115.

8. Flanagan CA, Becker, II, Davidson JS, Wakefield IK, Zhou W, Sealfon SC, et al. Glutamate 301 of the mouse gonadotropin-releasing hormone receptor confers specificity for arginine 8 of mammalian gonadotropin-releasing hormone. *J Biol Chem* (1994) **269**(36):22636-41.

9. Soderlund D, Canto P, de la Chesnaye E, Ulloa-Aguirre A, Mendez JP. A novel homozygous mutation in the second transmembrane domain of the gonadotrophin releasing hormone receptor gene. *Clin Endocrinol (Oxf)* (2001) **54**(4):493-8.

10. Conn PM, Leanos-Miranda A, Janovick JA. Protein origami: therapeutic rescue of misfolded geneproducts. *Molecular Interventions* (2002) **2**(5):308-16.

11. Janovick JA, Maya-Nunez G, Conn PM. Rescue of hypogonadotropic hypogonadism-causing and manufactured GnRH receptor mutants by a specific protein-folding template: misrouted proteins as a novel disease etiology and therapeutic target. *J Clin Endocrinol Metab* (2002) **87**(7):3255-62.

12. Marcos S, Sarfati J, Leroy C, Fouveaut C, Parent P, Metz C, et al. The prevalence of CHD7 missense versus truncating mutations is higher in patients with Kallmann syndrome than in typical CHARGE patients. *J Clin Endocrinol Metab* (2014) **99**(10):E2138-43. doi: 10.1210/jc.2014-2110. PubMed PMID: 25077900.

13. Betz SF, Reinhart GJ, Lio FM, Chen C, Struthers RS. Overlapping, nonidentical binding sites of different classes of nonpeptide antagonists for the human gonadotropin-releasing hormone receptor. *J Med Chem* (2006) **49**(2):637-47. PubMed PMID: 16420049.

14. Leanos-Miranda A, Janovick JA, Conn PM. Receptor-misrouting: an unexpectedly prevalent and rescuable etiology in gonadotropin-releasing hormone receptor-mediated hypogonadotropic hypogonadism. *J Clin Endocrinol Metab* (2002) **87**(10):4825-8.

15. Caron P, Chauvin S, Christin-Maitre S, Bennet A, Lahlou N, Counis R, et al. Resistance of hypogonadic patients with mutated GnRH receptor genes to pulsatile GnRH administration. *J Clin Endocrinol Metab* (1999) **84**(3):990-6.

16. Lu ZL, Gallagher R, Sellar R, Coetsee M, Millar RP. Mutations remote from the human gonadotropin-releasing hormone (GnRH) receptor-binding sites specifically increase binding affinity for GnRH II but not GnRH I: evidence for ligand-selective, receptor-active conformations. *J Biol Chem* (2005) **280**(33):29796-803. PubMed PMID: 15967801.

17. Ballesteros J, Kitanovic S, Guarnieri F, Davies P, Fromme BJ, Konvicka K, et al. Functional microdomains in G-protein-coupled receptors. The conserved arginine-cage motif in the gonadotropin-releasing hormone receptor. *J Biol Chem* (1998) **273**(17):10445-53.

18. Arora KK, Cheng Z, Catt KJ. Mutations of the conserved DRS motif in the second intracellular loop of the gonadotropin-releasing hormone receptor affect expression, activation, and internalization. *Mol Endocrinol* (1997) **11**(9):1203-12.

19. Arora KK, Sakai A, Catt KJ. Effects of second intracellular loop mutations on signal transduction and internalization of the gonadotropin-releasing hormone receptor. *J Biol Chem* (1995) **270**(39):22820-6.

20. Karges B, Karges W, Mine M, Ludwig L, Kuhne R, Milgrom E, et al. Mutation Ala(171)Thr stabilizes the gonadotropin-releasing hormone receptor in its inactive conformation, causing familial hypogonadotropic hypogonadism. *J Clin Endocrinol Metab* (2003) **88**(4):1873-9. PubMed PMID: 12679486.

21. Karges B, Karges W, de Roux N. Clinical and molecular genetics of the human GnRH receptor. *Hum Reprod Update* (2003) **9**(6):523-30. PubMed PMID: 14714589.

22. Myburgh DB, Pawson AJ, Davidson JS, Flanagan CA, Millar RP, Hapgood JP. A single amino acid substitution in transmembrane helix VI results in overexpression of the human GnRH receptor. *Eur J Endocrinol* (1998) **139**(4):438-47.

23. Bedecarrats GY, Linher KD, Janovick JA, Beranova M, Kada F, Seminara SB, et al. Four naturally occurring mutations in the human GnRH receptor affect ligand binding and receptor function. *Mol Cell Endocrinol* (2003) **205**(1-2):51-64. PubMed PMID: 12890567.

24. Lu Z-L, Coetsee M, White CD, Millar RP. Structural Determinants for Ligand-Receptor Conformational Selection in a Peptide G Protein-coupled Receptor. *J Biol Chem* (2007) **282**(24):17921-9.

25. Coetsee M, Gallagher R, Millar R, Flanagan C, Lu ZL. Role of Trp280(6.48) in the Gonadotropin-releasing hormone (GnRH) receptor. *Abstrct, BioScience2006, Glasgow UK* (2006).

26. Chauvin S, Berault A, Lerrant Y, Hibert M, Counis R. Functional importance of transmembrane helix 6 Trp(279) and exoloop 3 Val(299) of rat gonadotropin-releasing hormone receptor. *Mol Pharmacol* (2000) **57**(3):625-33.

27. Hovelmann S, Hoffmann SH, Kuhne R, ter Laak T, Reilander H, Beckers T. Impact of aromatic residues within transmembrane helix 6 of the human gonadotropin-releasing hormone receptor upon agonist and antagonist binding. *Biochemistry* (2002) **41**(4):1129-36.

28. Beneduzzi D, Trarbach EB, Latronico AC, Mendonca BB, Silveira LF. Novel mutation in the gonadotropin-releasing hormone receptor (GNRHR) gene in a patient with normosmic

isolated hypogonadotropic hypogonadism. *Arq Bras Endocrinol Metabol* (2012) **56**(8):540-4. PubMed PMID: 23295295.

29. Beneduzzi D, Trarbach EB, Min L, Jorge AA, Garmes HM, Renk AC, et al. Role of gonadotropin-releasing hormone receptor mutations in patients with a wide spectrum of pubertal delay. *Fertil Steril* (2014) **102**(3):838-46 e2. doi: 10.1016/j.fertnstert.2014.05.044. PubMed PMID: 25016926; PubMed Central PMCID: PMC4149947.

30. Layman LC, McDonough PG, Cohen DP, Maddox M, Tho SP, Reindollar RH. Familial gonadotropin-releasing hormone resistance and hypogonadotropic hypogonadism in a family with multiple affected individuals. *Fertil Steril* (2001) **75**(6):1148-55.

31. Tello JA, Newton CL, Bouligand J, Guiochon-Mantel A, Millar RP, Young J. Congenital hypogonadotropic hypogonadism due to GnRH receptor mutations in three brothers reveal sites affecting conformation and coupling. *PLoS One* (2012) **7**(6):e38456. doi: 10.1371/journal.pone.0038456. PubMed PMID: 22679506; PubMed Central PMCID: PMC3367945.

32. Meysing AU, Kanasaki H, Bedecarrats GY, Acierno JS, Jr., Conn PM, Martin KA, et al. GNRHR mutations in a woman with idiopathic hypogonadotropic hypogonadism highlight the differential sensitivity of luteinizing hormone and follicle-stimulating hormone to gonadotropin-releasing hormone. *J Clin Endocrinol Metab* (2004) **89**(7):3189-98. PubMed PMID: 15240592.

33. Isberg V, Mordalski S, Munk C, Rataj K, Harpsoe K, Hauser AS, et al. GPCRdb: an information system for G protein-coupled receptors. *Nucleic Acids Res* (2016) **44**(D1):D356-64. doi: 10.1093/nar/gkv1178. PubMed PMID: 26582914; PubMed Central PMCID: PMC4702843.

34. Pettersen EF, Goddard TD, Huang CC, Couch GS, Greenblatt DM, Meng EC, et al. UCSF Chimera--a visualization system for exploratory research and analysis. *J Comput Chem* (2004) **25**(13):1605-12. doi: 10.1002/jcc.20084. PubMed PMID: 15264254.

35. Forfar R, Lu ZL. Role of the transmembrane domain 4/extracellular loop 2 junction of the human gonadotropin-releasing hormone receptor in ligand binding and receptor conformational selection. *J Biol Chem* (2011) **286**(40):34617-26. doi: 10.1074/jbc.M111.240341. PubMed PMID: 21832286; PubMed Central PMCID: PMC3186411.

36. Millar RP, Pawson AJ, Morgan K, Rissman EF, Lu ZL. Diversity of actions of GnRHs mediated by ligand-induced selective signaling. *Front Neuroendocrinol* (2008) **29**(1):17-35. doi: 10.1016/j.yfrne.2007.06.002. PubMed PMID: 17976709; PubMed Central PMCID: PMC2667102.

37. Mayevu NM, Choe H, Abagyan R, Seong JY, Millar RP, Katz AA, et al. Histidine in the conserved peptide receptor activation domain of the gonadotropin releasing hormone receptor couples peptide binding and receptor activation. *Mol Cell Endocrinol* (2015) **402**:95-106. doi: 10.1016/j.mce.2015.01.008. PubMed PMID: 25583361.

38. Soderhall JA, Polymeropoulos EE, Paulini K, Gunther E, Kuhne R. Antagonist and agonist binding models of the human gonadotropin-releasing hormone receptor. *Biochem Biophys Res Commun* (2005) **333**(2):568-82. PubMed PMID: 15950933.

39. Millar RP, Lu ZL, Pawson AJ, Flanagan CA, Morgan K, Maudsley SR. Gonadotropin-releasing hormone receptors. *Endocr Rev* (2004) **25**(2):235-75. PubMed PMID: 15082521.

40. Stewart AJ, Sellar R, Wilson DJ, Millar RP, Lu ZL. Identification of a novel ligand binding residue Arg38(1.35) in the human gonadotropin-releasing hormone receptor. *Mol Pharmacol* (2008) **73**(1):75-81. doi: 10.1124/mol.107.040816. PubMed PMID: 17942747.

41. Davidson JS, McArdle CA, Davies P, Elario R, Flanagan CA, Millar RP. Asn102 of the gonadotropin-releasing hormone receptor is a critical determinant of potency for agonists containing C-terminal glycinamide. *J Biol Chem* (1996) **271**(26):15510-4.

42. Flanagan CA, Rodic R, Konvicka K, Yuen T, Chi L, Rivier JE, et al. Specific interactions of the Asp2.61(98) side chain of the gonadotropin-releasing hormone receptor contribute differentially to ligand interaction and receptor expression. *Biochemistry* (2000) **39**:8133-41.

43. Coetsee M, Millar RP, Flanagan CA, Lu ZL. Identification of Tyr(290(6.58)) of the human gonadotropin-releasing hormone (GnRH) receptor as a contact residue for both GnRH I and GnRH II: importance for high-affinity binding and receptor activation. *Biochemistry* (2008) **47**(39):10305-13. PubMed PMID: 18771291.

44. Fromme BJ, Katz AA, Roeske RW, Millar RP, Flanagan CA. Role of aspartate7.32(302) of the human gonadotropin-releasing hormone receptor in stabilizing a high-affinity ligand conformation. *Mol Pharmacol* (2001) **60**(6):1280-7.
